# The efficacy of core decompression combined with regenerative therapy in early femoral head necrosis: a systematic review and meta-analysis involving 954 subjects

**DOI:** 10.3389/fphar.2024.1501590

**Published:** 2025-01-07

**Authors:** Haiwei Tang, Tingxian Ling, Enze Zhao, Mingke You, Xi Chen, Gang Chen, Kai Zhou, Zongke Zhou

**Affiliations:** Department of Orthopedics, West China Hospital, Sichuan University, Chengdu, Sichuan, China

**Keywords:** osteonecrosis of the femoral head, core decompression, regenerative therapy, joint preservation surgery, meta-analysis

## Abstract

**Background:**

The debate continues on whether combining core decompression (CD) with regenerative therapy provides a more effective treatment for early femoral head necrosis than CD alone. This systematic review and meta-analysis endeavored to assess its efficacy.

**Methods:**

We systematically searched PubMed, Web of Science, and Cochrane Library through July 2024 for RCTs and cohort studies evaluating the impact of core decompression (CD) with regenerative therapy versus CD alone in early-stage osteonecrosis (ARCO I, II or IIIa or Ficat I or II) of the femoral head (ONFH). Bias was evaluated using the Cochrane ROB 2.0 for RCTs and the Newcastle-Ottawa Scale (NOS) for cohort studies. The primary outcome was disease progression, measured by the incidence of staging advancement and total hip arthroplasty (THA) conversion. Clinical outcomes, including VAS, HHS, WOMAC, and Lequesne index, were secondary measures. Subgroup analyses were performed for variables such as age, BMI, follow-up period, and dosage in the bone marrow aspirate concentrate (BMAC) group, with results depicted in forest plots.

**Results:**

This study represented a total of seven RCTs (mean follow-up time 36.57 months) and eight cohort trials (mean follow-up time 74.18 months) involving 954 hips. CD, when combined with agents, exhibited considerably enhanced efficacy over CD alone (risk ratio (RR) = 0.55 (95% CI 0.39–0.77), *p* < 0.001, *I* ^*2*^ = 54%) and 0.59 (95% CI 0.43–0.81), *p* = 0.001, *I* ^*2*^ = 51%), respectively). However, a significant difference was exclusive to the CD combined with BMAC group in terms of stage progression outcomes (stage progression, RR = 0.47 (95% CI 0.28–0.78), *p* = 0.004, *I* ^*2*^ = 67%); THA conversions, RR = 0.41 (95% CI 0.32–0.52), *p* < 0.001, *I* ^*2*^ = 43%). Secondary outcomes (VAS, HHS, WOMAC score and Lequesne index) showed improved results when CD was combined with other regenerative agents, such as bone mesenchymal stem cells (BMSCs) and bone morphogenetic proteins (BMPs), etc. In the reported data, the regenerative group demonstrated significantly higher rates of subjective improvement in pain and functional outcomes compared to those in the CD group (71.74% (66/92) vs. 56.38% (53/94). Subgroup analysis revealed superior outcomes in the low-dose (less than 20 mL) BMAC group and patients aged under 40 years old in stage progression rate and THA conversion rate.

**Conclusion:**

CD, when combined with regenerative therapy, can diminish hip pain and enhance functionality, but its ability to slow disease progression remains uncertain. BMAC presents a more substantiated efficacy evidence than other agents, with low-doses of BMAC in patients under 40 years potentially slowing ONFH progression. Nonetheless, the high heterogeneity and relatively short follow-up time of these studies make it difficult to draw accurate conclusions, which necessitates verification through future trials comparing CD versus CD combined with regenerative therapy, with a focus on extended follow-up periods.

**Systematic Review Registration:**

identifier CRD42023467873.

## Introduction

Osteonecrosis of the femoral head (ONFH) is a pathological condition characterized by the localized demise of osteocytes and bone marrow elements, attributable to compromised arterial perfusion, venous stasis, or structural disruption of the femoral head (Wen et al., 2022). As a common and refractory disease in orthopedics, ONFH results in a huge economic burden worldwide. In the United States, the condition affects over 10,000 new patients annually and contributes to 10% of all hip arthroplasty (THA) (Mont et al., 2015). In Japan, the annual incidence rate was 1.91 per 100,000, which was estimated that there were around 2,400 cases per year from 2010 to 2013 (Ikeuchi et al., 2015). And the cumulative number of ONFH patients up to 8.12 million in 2013 in China (Zhao et al., 2015). The etiology of ONFH is multifactorial and individual-specific, which can be divided into two major categories: traumatic and nontraumatic. Major etiological factors of traumatic ONFH include femoral neck fracture, acetabular fracture, femoral head dislocation, and severe hip sprain or contusion, while nontraumatic ONFH is triggered by application of corticosteroid (the most common type), excessive alcohol consumption, decompression sickness, hemoglobin disease, autoimmune diseases (like systematic lupus erythematosus) and idiopathic diseases (Mont et al., 2020; Zhao et al., 2020; Zheng et al., 2022). Thus, heavy corticosteroid use and alcohol abuse are risk factors for ONFH. Additionally, smoking and obesity are also associated with an increased risk (Cao et al., 2016; Takahashi et al., 2012). While THA is the prevalent treatment for ONFH, there has been a notable shift towards head-preserving procedures, particularly core decompression (CD), especially favored for younger and more active demographics due to the anticipated need for a minimum of one revision post-THA, aseptic loosening and prosthesis wear (Mont et al., 2020; Ng et al., 2023). The fundamental rationale behind CD is to alleviate intramedullary pressure and bolster blood circulation, thereby fostering revascularization and osteogenesis at the affected site (Liu et al., 2018). Despite a consensus from a handful of small-scale randomized trials suggesting the superiority of CD over nonoperative interventions (Mont et al., 2020), the literature is replete with conflicting reports questioning the efficacy of CD in preventing femoral head collapse (Yoon et al., 2018; Chughtai et al., 2017). In a study of 1,206 hips CD patients, Mont et al. discovered that the necrosis of 36% of the cases continued to progress following CD alone (Mont et al., 1996). Hua et al. (2019) and Goodman (2000) also contended that mere CD is insufficient to halt the progression of ONFH, asserting that robust support for the subchondral bone is imperative. Moreover, CD’s success is significantly higher in Ficat stages I and II than in stage III, underscoring its applicability primarily in the early phases of ONFH (Hua et al., 2019).

Given the potential for iatrogenic collapse in the drilled region following CD, it is imperative to consider robust support mechanisms (Hua et al., 2019; Goodman, 2000). Due to the limited availability of autologous or allogeneic bone grafts, as well as concerns regarding donor complications and immune rejection, there is a pressing need for innovative methodologies (Tang et al., 2024). Hernigou et al. (1999) demonstrated that both the quantity and functionality of bone mesenchymal stem cells (BMSCs) were diminished in patients with ONFH, thereby offering novel insights for researchers aiming to adjust the pathological microenvironment and facilitate bone regeneration through exogenous supplementation of these cells, ultimately providing necessary support. In 2004, A seminal study by Gangji et al. (2004) compared the standalone efficacy of CD to CD combined with bone marrow aspirate concentrate (BMAC), revealing that the latter combination was more effective in preventing collapse and ameliorating symptoms, which aroused wide concern. Therefore, BMAC may enhance the efficacy of CD by enhancing osteogenic ability and regulating the bone marrow microenvironment (Calori et al., 2017). Recently, the advent of regenerative therapies such as BMSCs, bone morphogenetic proteins (BMPs), platelet-rich plasma (PRP), and osteoblasts (OB) has spurred the integration of these modalities with CD, presenting a promising strategy for ONFH management. However, the synergistic effects of combining CD with regenerative therapies are still under scrutiny, with diverse treatment agents yielding varied outcomes (Wang et al., 2024; Hu et al., 2023; Liu et al., 2021; Wang et al., 2023).

This systematic review and meta-analysis aims to critically assess the efficacy of combining CD with regenerative therapies versus CD alone in the prevention of femoral head collapse and alleviation of symptoms in patients with precollapsing or mild collapsed ONFH (ARCO stage I, II and IIIa or Ficat stage I and II). Additionally, we seek to identify patient characteristics that may predict a favorable response to CD combined with regenerative therapies through subgroup analysis. We hypothesize that the cohort receiving CD in conjunction with a regenerative agent will demonstrate improved therapeutic outcomes, particularly in terms of disease stage progression and clinical manifestations. We anticipate that younger individuals with lower body mass indices (BMIs) will derive greater benefits from this combined therapeutic approach.

## Materials and methods

The present meta-analysis was conducted in adherence to the Preferred Reporting Items for Systematic Reviews and Meta-analysis (PRISMA) guidelines (Page et al., 2021) and Assessing of Multiple Systematic Reviews (AMSTAR) criteria (Shea et al., 2017). This study protocol was registered with PROSPERO (ID number: CRD42023467873), ensuring transparency and protocol adherence.

### Search strategy

A comprehensive literature searched was performed in PubMed, Web of Science, and the Cochrane Library databases from their inception through 19 July 2024. The search strategy incorporated the following search terms: “osteonecrosis of the femoral head,” “femoral head necrosis, ” “femur head necrosis,” “avascular necrosis in femoral head,” “avascular necrosis of the femoral head,” “cell therapy,” “regenerative therapy,” “regenerative therapies,” “regeneration therapy,” “BMAC,” “bone marrow aspirate concentrate,” “BMSCs,” “bone mesenchymal stem cells,” “MSCs,” “mesenchymal stem cells,” “PRP,” “platelet-rich plasma,” “OB,” “osteoblasts,” “core decompression,” and “CD.” No language restrictions were applied to the search.

### Eligibility criteria

Study selection was based on a thorough review of abstracts and full texts. Inclusion criteria were as follows: 1) Population: Patients with nontraumatic femoral head necrosis in the ARCO stage I, II and IIIa or Ficat stage I and II, aged 18 years or older; 2) Intervention: Patient receiving CD combined with any regenerative treatment; 3) Comparator: Patients received CD alone; 4) Outcomes: Primary outcomes included the number of progressions to severe collapse (defined as progressive collapse of ≥2 mm within the follow-up period) (Osawa et al., 2021) and THA conversion; secondary outcomes included clinical outcomes [visual analog scale (Li et al., 2013) score, Harris Hip Score [HHS] score, Western Ontario and McMaster Universities Osteoarthritis Index [WOMAC] score and Lequesne Index (Lequesne et al., 1987)]. 5) Study design: Randomized controlled trials (RCTs) or cohort studies with a control group were included. Exclusion criteria were: 1) Patients with traumatic femoral head necrosis or in a severe collapsed (>2 mm) phase; 2) Animal studies; 3) Noncomparative studies, case reports, and case series; 4) Nonoriginal research such as reviews and technical reports; 5) Studies from which relevant data could not be extracted, and those that did not respond to requests for data from the original authors.

### Data extraction

Data from eligible studies were extracted by two independent reviewers. The extracted data included the first author, publication year, study type, number of hips, patient characteristics, type of regenerative therapy, dosage of agents, follow-up duration, disease stage, and outcomes (number of hips progressing to collapse and THA conversion, VAS score, HHS score, WOMAC score, and Lequesne Index), excluding the late-stage cases (ARCO stage IIIb or IV). If the original text does not explicitly specify the precise value, specific data is extracted using Origin 2021 software from image. Graphical data were quantified using Plot Digitizer software (Version 2.6.8, Joseph Huwaldt and Scott Steinhorst) (Jelicic Kadic et al., 2016).

### Quality assessment

The risk of bias in RCTs was assessed using the revised Cochrane risk of bias tool for randomized trials (RoB 2.0) (Sterne et al., 2019) which evaluates randomization process, deviations from intended interventions, missing outcome data, measurement of the outcome, selection of the reported result and overall bias. For non-RCT trials, six additional criteria based on NOS scale were used to assess the potential of bias: selection bias, detection bias, comparability, bias in measurement of outcomes, bias due to missing data, and adequacy of follow-up.

### Outcome measures

In these included literature, the patients were all clinical visits during follow-up, and the researchers were responsible for data collection. The primary outcomes were the number of hips progressing to collapse and conversion to THA. Secondary outcomes included clinical outcomes. Subgroup analysis was conducted based on the type of regenerative agent, patient age, BMI, BMAC dosage, and mean follow-up duration (based on the mean statistical characteristics and follow-up time of the patients). Following Mao et al. (2020), patients were divided into subgroups based on age (under 40 years old being the ideal age group for stem cell therapy). Additionally, based on a retrospective study (Pan et al., 2020), patients were categorized based on BMI (greater than 24 years old being associated with a higher risk of joint-preservation failure). BMAC dosage were artificially divided into three groups: low (less than 20 mL), medium (20–40 mL), and high (more than 40 mL), considering the approximate volume of ARCO stage III or IV femoral head necrosis (22 cm^3^) (Hu et al., 2015). The mean follow-up duration was categorized into three subgroups: less than 24 months, 24–60 months, and more than 60 months.

### Statistical analysis

All statistical analyses were performed using RevMan 5.4.1 software. Continuous data were presented as mean difference (MD) with 95% confidence interval (CI), while binary data were presented as risk ratio (RR) with 95% CI. Heterogeneity was assessed using the *I* ^*2*^ statistic with a value of 50% or higher indicating higher significant heterogeneity. The random effects model assumes that not only the effects vary across different studies, but also that their underlying true effects are drawn from a specific distribution. Consequently, it is capable of accommodating variations both within and between studies (Halme et al., 2023). Thus, the random effect model was used for *I* ^*2*^ > 50%, and the fixed effect model was used for *I* ^*2*^ < 50%. Subgroup analyses were conducted to compare efficacy of different BMAC dosage, age groups, BMIs and follow-up durations. Funnel plots were used to detect publication bias, and a *p* < 0.05 was considered statistically significant.

## Results

### Selection of included studies

**FIGURE 1:**
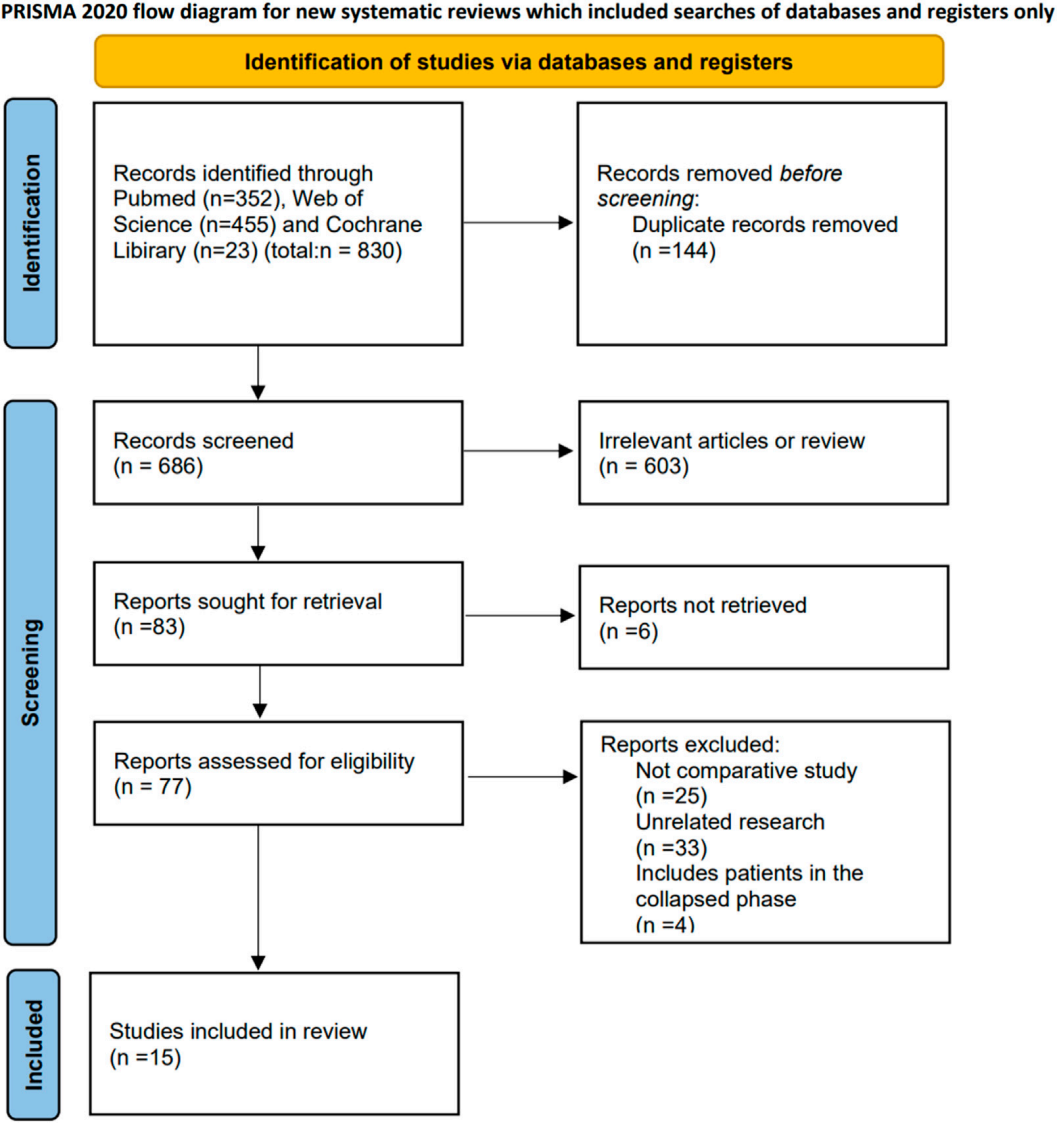
Flow chart of the search and search results.

The systematic search yielded a total of 830 articles, comprising 352 from the PubMed database and 478 from other databases, with duplicate articles subsequently removed. A total of 603 irrelevant articles were removed after the title and abstract were checked. Finally, 15 publications were eventually included in the study after the full-text reviews. The included studies compared CD alone versus CD combined with various regenerative therapies, including BMAC (Gangji et al., 2004; Boontanapibul et al., 2021; Cruz-Pardos et al., 2016; Gangji et al., 2011; Hernigou et al., 2018; Pepke et al., 2016; Tabatabaee et al., 2015), BMSCs (Kang et al., 2018; Nally et al., 2018), PRP (Aggarwal et al., 2021) and OB ect. (Jayankura et al., 2023; Liang et al., 2023; Martinot et al., 2020). Additionally, two studies compared CD combined with BMAC versus OB (Gangji et al., 2016; Hauzeur et al., 2020). In two of these studies, bone plugs were employed to seal the inlet of the pipe subsequent to injection in order to avert leakage of the regenerated agent (Tabatabaee et al., 2015; Aggarwal et al., 2021). To ensure the reliability of the results, we conducted subgroup analyses of regenerative agent therapies utilizing this specific approach as opposed to conventional regenerative agent therapies for the purpose of evaluating efficacy; Additionally, there was a study that incorporated regeneration agents with iliac bone particles into the channel, and thus it was excluded (Fu et al., 2022). Tables 1, 2 illustrate the characteristics of the included studies, and the study selection process is depicted in Figure 1.

**TABLE 1:** Characteristics of included studies.

Study	Design	Level of evidence^a^	Intervention	Control (Drill diameter)	Dosage of agents	NO. of Hips (C/T)	Age (C/T) (SD)	BMI (Kg/m^2^) (C/T) (SD)	Follow-up (month)	Disease Stage	Outcomes Primary Secondary	
Gangji et al. (2011)	Prospective, RCT	Ib	CD + BMAC	CD (NR)	49.7 mL	11/13	45.7 (2.8)/42.2 (2.6)	NR	60 m	ACRO Ⅰ/Ⅱ	Progression to collapse, THA conversion	VAS, Lequesne Index
Tabatabaee et al. (2015)	Prospective RCT	Ib	CD + BMAC (bone plugs)	CD (2.7 mm)	58 mL	9/12	26.8 (5.8)/31 (11.4)	NR	24 m	ACRO Ⅰ/Ⅱ	Progression to collapse, THA conversion	VAS, WOMAC
Pepke et al. (2016)	Prospective RCT	Ib	CD + BMAC	CD (5 mm)	10 mL	14/11	44.5 (3.3)/44.3 (3.4)	NR	24 m	ACRO Ⅰ/Ⅱ	THA conversion	VAS HHS
Aggarwal et al. (2021)	Prospective RCT	Ib	CD + PRP (bone plugs)	CD (10 mm)	8 mL	28/25	35.2 (12.5)/38.2 (10.4)	NR	64 m	Ficat I/II	Progression to collapse, THA conversion	HHS
Jayankura et al. (2023)	Prospective RCT	Ib	CD + OB	CD (NR)	5 mL	29/25	45 (10)/46 (10)	NR	12 m	ACRO Ⅰ/Ⅱ	Progression to collapse, THA conversion	HHS
Hauzeur et al. (2020)	Prospective RCT	Ib	CD + OB	CD + BMAC (3 mm)	40 mL	26/27	50 (12)/51 (10)	27 (5)/26 (5)	36 m	ACRO Ⅰ/Ⅱ	Progression to collapse, THA conversion	—
Gangji et al. (2016)	Prospective RCT	Ib	CD + OB	CD + BMAC (NR)	41 mL	30/30	50.6 (11.8)/50.6 (11.8)	NR	36 m	ACRO Ⅰ/Ⅱ	Progression to collapse	—
Boontanapibul et al. (2021)	Retrospective cohort	IIb	CD + BMAC	CD (3.2 mm)	5 mL	33/50	43 (10)/38 (13)	28 (6)/27 (5)	36 m	ACRO Ⅰ/Ⅱ/IIIa	Progression to collapse, THA conversion	—
Hernigou et al. (2018)	Prospective cohort	IIb	CD + BMAC	CD (4 mm)	20 mL	125/125	36 (7)/36 (7)	NR	300 m	ACRO Ⅰ/Ⅱ	Progression to collapse, THA conversion	VAS, HHS, WOMAC
Cruz-Pardos et al. (2016)	Retrospective cohort	IIb	CD + BMAC	CD (4 mm)	20 mL	19/41	38.9 (13)/43.3 (10.8)	NR	45 m	Ficat I/II	Progression to collapse, THA conversion	—
Gangji et al. (2004)	Prospective cohort	IIb	CD + BMAC	CD (3 mm)	51 mL	8/10	48.8 (11.2)/40.9 (9.8)	NR	24 m	ACRO Ⅰ/Ⅱ	Progression to collapse, THA conversion	VAS, WOMAC, Lequesne Index
Kang et al. (2018)	Retrospective cohort	IIb	CD + BMSCs	CD (NR)	15 mL	30/30	47.3 (9.7)/46.0 (9.3)	24.0 (4.1)/23.8 (3.7)	51.36 m	ACRO Ⅰ/Ⅱ	Progression to collapse, THA conversion	—
Nally et al. (2018)	Retrospective cohort	IIb	CD + BMSCs	CD (7 mm)	NR	47/16	38.9 (10.1)/40.4 (12.8)	NR	72 m	Ficat I/II	THA conversion	—
Liang et al. (2023)	Retrospective cohort	IIb	CD + BMMC + PRP	CD (3 mm)	7 mL	20/24	37.5 (5.3)/36.4 (5.3)	25.59 (3.43)/25.11 (2.89)	41.1 m	ACRO Ⅰ/Ⅱ/IIIa	Progression to collapse, THA conversion	VAS, HHS
Martinot et al. (2020)	Retrospective cohort	IIb	CD + BM	CD (4.3 mm)	NR	23/40	41.06 (9.53)/43.23 (10.97)	26.27 (3.78)/27.24 (4.13)	24 m	Ficat I/II	THA conversion	HHS
Martinot et al. (2020)	Retrospective cohort	IIb	CD + BM + BMP	CD (4.3 mm)	NR	23/23	41.06 (9.53)/37.47 (10.10)	26.27 (3.78)/25.31 (4.17)	24 m	Ficat I/II	THA conversion	HHS

T, intervention group; C, control group; RCT, randomized clinical trial; CD, core decompression; BMAC, bone marrow aspirate concentrate; PRP, platelet-rich plasma; OB, osteoblast; BMSCs, bone marrow mesenchymal stem cells; BMMC, bone marrow mononuclear cell; BM, bone marrow; BMP, bone-morphogenetic protein; VAS, visual analog scale; HHS, harris hip score; WOMAC, Western Ontario and McMaster Universities Osteoarthritis Index; ARCO, association research circulation osseous; NR, not report.

^a^ Oxford Centre for Evidence-based Medicine.

**TABLE 2:** Details of the outcomes of CD + BMAC vs. CD.

Outcomes	No. of studies	No. of hips	Statistical method	Pooled method	*I* ^*2*^	Effect size	*p*
Staging advancement							
1 Progression to collapse							
1.1 BMAC dose							
High dose	31 (Takahashi et al., 2012; Gangji et al., 2011; Tabatabaee et al., 2015)	63 (35 vs. 28)	RR (95% CI)	Fix, M-H	0%	0.19 [0.08, 0.46]	<0.001
Medium dose	2 (Cruz-Pardos et al., 2016; Hernigou et al., 2018)	310 (166 vs. 144)	RR (95% CI)	Random, M-H	90%	0.62 [0.24, 1.59]	0.320
Low dose	1 (Boontanapibul et al., 2021)	83 (50 vs. 33)	RR (95% CI)	Fix, M-H	NA	0.56 [0.34, 0.92]	0.020
Overall	6 (Gangji et al., 2004; Boontanapibul et al., 2021; Cruz-Pardos et al., 2016; Gangji et al., 2011; Hernigou et al., 2018; Tabatabaee et al., 2015)	456 (251 vs. 205)	RR (95% CI)	Random, M-H	67%	0.47 [0.28, 0.78]	0.004
1.2 Age							
Age >40	3 (Gangji et al., 2004; Cruz-Pardos et al., 2016; Gangji et al., 2011)	102 (64 vs. 38)	RR (95% CI)	Random, M-H	71%	0.47 [0.15, 1.46]	0.190
Age <40	3 (Boontanapibul et al., 2021; Hernigou et al., 2018; Tabatabaee et al., 2015)	354 (187 vs. 167)	RR (95% CI)	Fix, M-H	44%	0.40 [0.31, 0.52]	<0.001
Overall	6 (Gangji et al., 2004; Boontanapibul et al., 2021; Cruz-Pardos et al., 2016; Gangji et al., 2011; Hernigou et al., 2018; Tabatabaee et al., 2015)	456 (251 vs. 205)	RR (95% CI)	Random, M-H	67%	0.47 [0.28, 0.78]	0.004
1.3 Follow-up time							
<24 months	2 (Gangji et al., 2004; Tabatabaee et al., 2015)	39 (22 vs. 17)	RR (95% CI)	Fix, M-H	0%	0.10 [0.02, 0.50]	0.005
24–60 months	3 (Boontanapibul et al., 2021; Cruz-Pardos et al., 2016; Gangji et al., 2011)	167 (104 vs. 63)	RR (95% CI)	Random, M-H	61%	0.63 [0.35, 1.14]	0.130
>60 months	1 (Hernigou et al., 2018)	250 (125 vs. 125)	RR (95% CI)	Fix, M-H	NA	0.39 [0.29, 0.53]	<0.001
Overall	6 (Gangji et al., 2004; Boontanapibul et al., 2021; Cruz-Pardos et al., 2016; Gangji et al., 2011; Hernigou et al., 2018; Tabatabaee et al., 2015)	456 (251 vs. 205)	RR (95% CI)	Random, M-H	67%	0.47 [0.28, 0.78]	0.004
2 THA conversion							
2.1 BMAC dose							
High dose	3 (Gangji et al., 2004; Gangji et al., 2011; Tabatabaee et al., 2015)	63 (35 vs. 28)	RR (95% CI)	Fix, M-H	0%	0.35 [0.10, 1.23]	0.100
Medium dose	2 (Cruz-Pardos et al., 2016; Hernigou et al., 2018)	310 (166 vs. 144)	RR (95% CI)	Random, M-H	86%	0.50 [0.19, 1.35]	0.170
Low dose	2 (Boontanapibul et al., 2021; Pepke et al., 2016)	108 (61 vs. 47)	RR (95% CI)	Fix, M-H	0%	0.55 [0.35, 0.88]	0.010
Overall	7 (Gangji et al., 2004; Boontanapibul et al., 2021; Cruz-Pardos et al., 2016; Gangji et al., 2011; Hernigou et al., 2018; Pepke et al., 2016; Tabatabaee et al., 2015)	481 (262 vs. 219)	RR (95% CI)	Fix, M-H	43%	0.41 [0.32, 0.52]	<0.001
2.2 Age							
Age >40	4 (Gangji et al., 2004; Cruz-Pardos et al., 2016; Gangji et al., 2011; Pepke et al., 2016)	127 (75 vs. 52)	RR (95% CI)	Fix, M-H	0%	0.73 [0.44, 1.22]	0.230
Age <40	3 (Boontanapibul et al., 2021; Hernigou et al., 2018; Tabatabaee et al., 2015)	354 (187 vs. 167)	RR (95% CI)	Fix, M-H	0%	0.35 [0.26, 0.46]	<0.001
Overall	7 (Gangji et al., 2004; Boontanapibul et al., 2021; Cruz-Pardos et al., 2016; Gangji et al., 2011; Hernigou et al., 2018; Pepke et al., 2016; Tabatabaee et al., 2015)	481 (262 vs. 219)	RR (95% CI)	Fix, M-H	43%	0.41 [0.32, 0.52]	<0.001
2.3 Follow-up time							
<24 months	3 (Gangji et al., 2004; Pepke et al., 2016; Tabatabaee et al., 2015)	64 (33 vs. 31)	RR (95% CI)	Fix, M-H	0%	0.55 [0.23, 1.33]	0.190
24–60 months	3 (Boontanapibul et al., 2021; Cruz-Pardos et al., 2016; Gangji et al., 2011)	167 (104 vs. 63)	RR (95% CI)	Fix, M-H	0%	0.61 [0.41, 0.90]	0.010
>60 months	1 (Hernigou et al., 2018)	250 (125 vs. 125)	RR (95% CI)	Fix, M-H	NA	0.32 [0.23, 0.44]	<0.001
Overall	7 (Gangji et al., 2004; Boontanapibul et al., 2021; Cruz-Pardos et al., 2016; Gangji et al., 2011; Hernigou et al., 2018; Pepke et al., 2016; Tabatabaee et al., 2015)	481 (262 vs. 219)	RR (95% CI)	Fix, M-H	43%	0.41 [0.32, 0.52]	<0.001
2.4 BMI							
BMI >24	3 (Boontanapibul et al., 2021; Liang et al., 2023; Martinot et al., 2020)	236 (137 vs. 99)	RR (95% CI)	Fix, M-H	0%	0.55 [0.38, 0.77]	<0.001
BMI <24	1 (Kang et al., 2018)	60 (30 vs. 30)	RR (95% CI)	Fix, M-H	NA	0.40 [0.18, 0.89]	0.020
Overall	4 (Boontanapibul et al., 2021; Kang et al., 2018; Liang et al., 2023; Martinot et al., 2020)	296 (167 vs. 129)	RR (95% CI)	Fix, M-H	0%	0.51 [0.37, 0.71]	<0.001
Clinical outcomes							
1 VAS score							
CD + BMAC	4 (Gangji et al., 2004; Gangji et al., 2011; Hernigou et al., 2018; Pepke et al., 2016)	317 (159 vs. 158)	MD (95% CI)	Random, I-V	91%	−15.87 [−24.43, −7.31]	<0.001
CD + BMSCs	2 (Tabatabaee et al., 2015; Kang et al., 2018)	81 (42 vs. 39)	MD (95% CI)	Random, I-V	96%	−7.23 [−24.97,10.50]	0.420
CD + BMMC + PRP	1 (Liang et al., 2023)	44 (24 vs. 20)	MD (95% CI)	Fix, I-V	NA	−12.00 [−21.67, −2.33]	0.020
Overall	7 (Gangji et al., 2004; Gangji et al., 2011; Hernigou et al., 2018; Pepke et al., 2016; Tabatabaee et al., 2015; Kang et al., 2018; Liang et al., 2023)	442 (225 vs.217)	MD (95% CI)	Random, I-V	91%	−12.86 [−18.36, −7.36]	<0.001
2 HHS score							
CD + BMAC	2 (Hernigou et al., 2018; Pepke et al., 2016)	275 (136 vs. 139)	MD (95% CI)	Random, I-V	72%	8.99 [4.08, 13.90]	<0.001
CD + PRP	1 (Aggarwal et al., 2021)	53 (25 vs.28)	MD (95% CI)	Fix, I-V	NA	14.30 [8.42, 20.19]	<0.001
CD + BMMC + PRP	1 (Liang et al., 2023)	44 (24 vs. 20)	MD (95% CI)	Fix, I-V	NA	5.73 [1.60, 9.86]	0.007
Overall	4 (Hernigou et al., 2018; Pepke et al., 2016; Aggarwal et al., 2021; Liang et al., 2023)	372 (185 vs. 187)	MD (95% CI)	Random, I-V	72%	9.19 [5.69, 12.70]	<0.001
3 WOMAC score							
CD + BMAC	3 (Gangji et al., 2004; Hernigou et al., 2018; Tabatabaee et al., 2015)	289 (147 vs. 142)	MD (95% CI)	Random, I-V	98%	−10.78 [−21.08, −0.47]	0.040
CD + OB	1 (Jayankura et al., 2023)	44 (21 vs. 23)	MD (95% CI)	Fix, I-V	NA	4.00 [−11.09, 19.09]	0.600
Overall	4 (Gangji et al., 2004; Hernigou et al., 2018; Tabatabaee et al., 2015; Jayankura et al., 2023)	333 (168 vs. 165)	MD (95% CI)	Random, I-V	97%	−8.34 [−17.66, 0.98]	0.080
4 Lequesne Index							
CD + BMAC	2 (Gangji et al., 2004; Gangji et al., 2011)	42 (23 vs. 19)	MD (95% CI)	Random, I-V	63%	−3.39 [−4.96, −1.83]	<0.001

CD, core decompression; BMAC, bone marrow aspirate concentrate; PRP, platelet rich plasma; OB, osteoblast; BMSCs, bone marrow mesenchymal stem cells; BMMC, bone marrow mononuclear cell; VAS, visual analog scale; HHS, harris hip score; WOMAC, Western Ontario and McMaster Universities Osteoarthritis Index; CI, confidence interval; RR, relative risk; MD, mean difference; I–V, Inverse–Variance; M–H, Mantel–Haenszel. NA, not applicable.

### Study characteristics

Our meta-analysis encompassed 15 studies that evaluated the efficacy of CD versus CD combined with regenerative therapies in 954 hip lesions. The majority of the studies involved sample sizes exceeding 20 hips (Boontanapibul et al., 2021; Cruz-Pardos et al., 2016; Gangji et al., 2011; Hernigou et al., 2018; Pepke et al., 2016; Tabatabaee et al., 2015; Kang et al., 2018; Nally et al., 2018; Aggarwal et al., 2021; Jayankura et al., 2023; Liang et al., 2023; Martinot et al., 2020; Gangji et al., 2016; Hauzeur et al., 2020), with a minimum follow-up of 12 months, an average patient age of 41.20 ± 11.13 and a mean BMI of 26.08 ± 4.60 kg/m^2^. No significant differences between patients receiving CD alone versus those receiving CD combined with regenerative therapies. Staging systems varied across studies, with four utilizing Ficat staging (Cruz-Pardos et al., 2016; Nally et al., 2018; Aggarwal et al., 2021; Martinot et al., 2020), and the remaining eleven employing ARCO staging (Gangji et al., 2004; Boontanapibul et al., 2021; Gangji et al., 2011; Hernigou et al., 2018; Pepke et al., 2016; Tabatabaee et al., 2015; Kang et al., 2018; Jayankura et al., 2023; Liang et al., 2023; Gangji et al., 2016; Hauzeur et al., 2020). Clinical outcomes are assessed using the number of hips with stage progression and THA conversion, as well as the Visual Analog Scale (VAS), Harris Hip Score (HHS), Western Ontario and McMaster Universities Osteoarthritis Index (WOMAC) and Lequesne Index. Detailed study characteristics are summarized in Table 1, with two rows allocated to one study (Martinot et al., 2020) that utilized two comparison groups.

### Assessment for risk of bias

**FIGURE 2:**
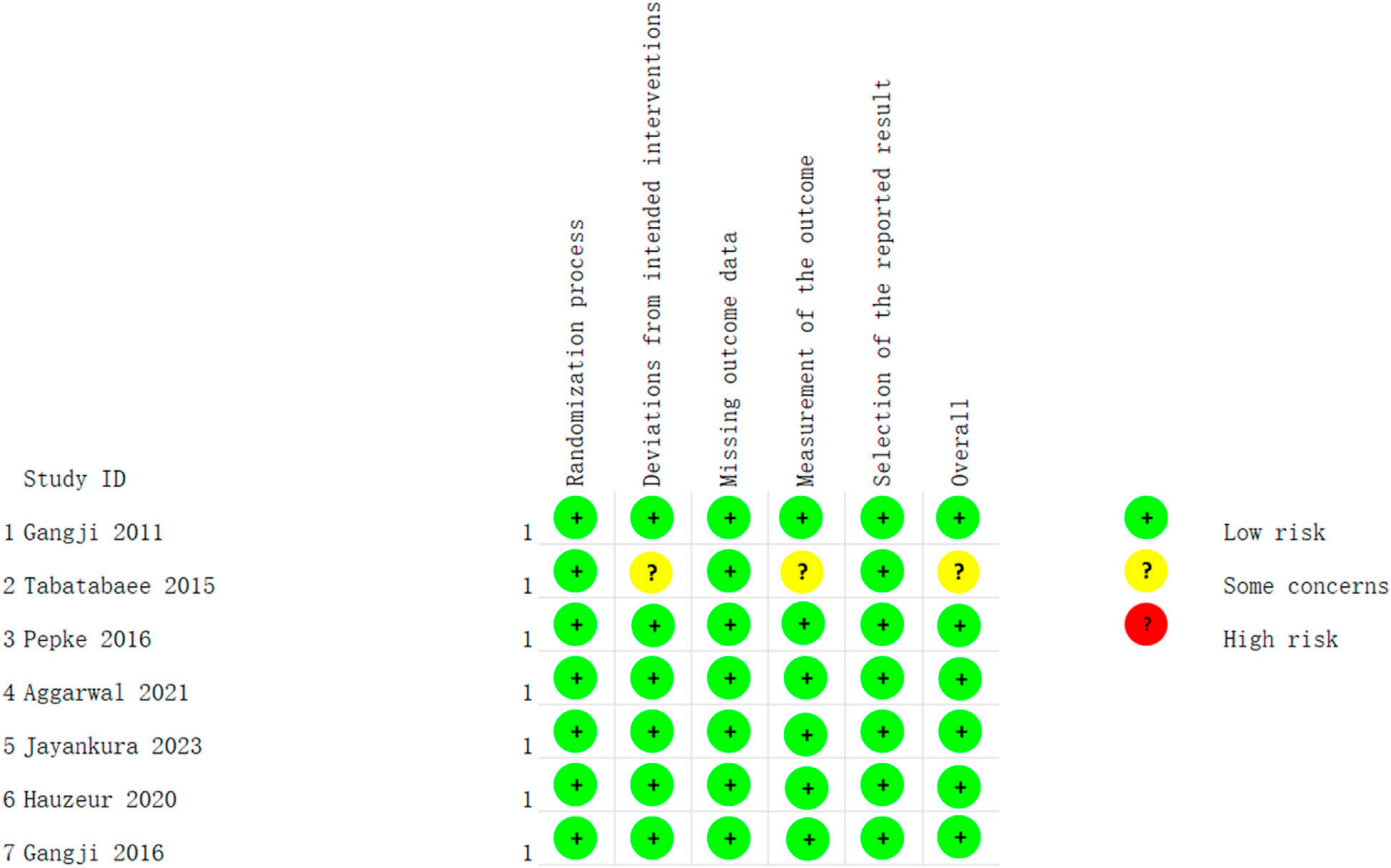
Risk of bias summary of RCTs: low risk of bias in green; some concerns of bias in yellow; high risk of bias in red.

**FIGURE 3:**
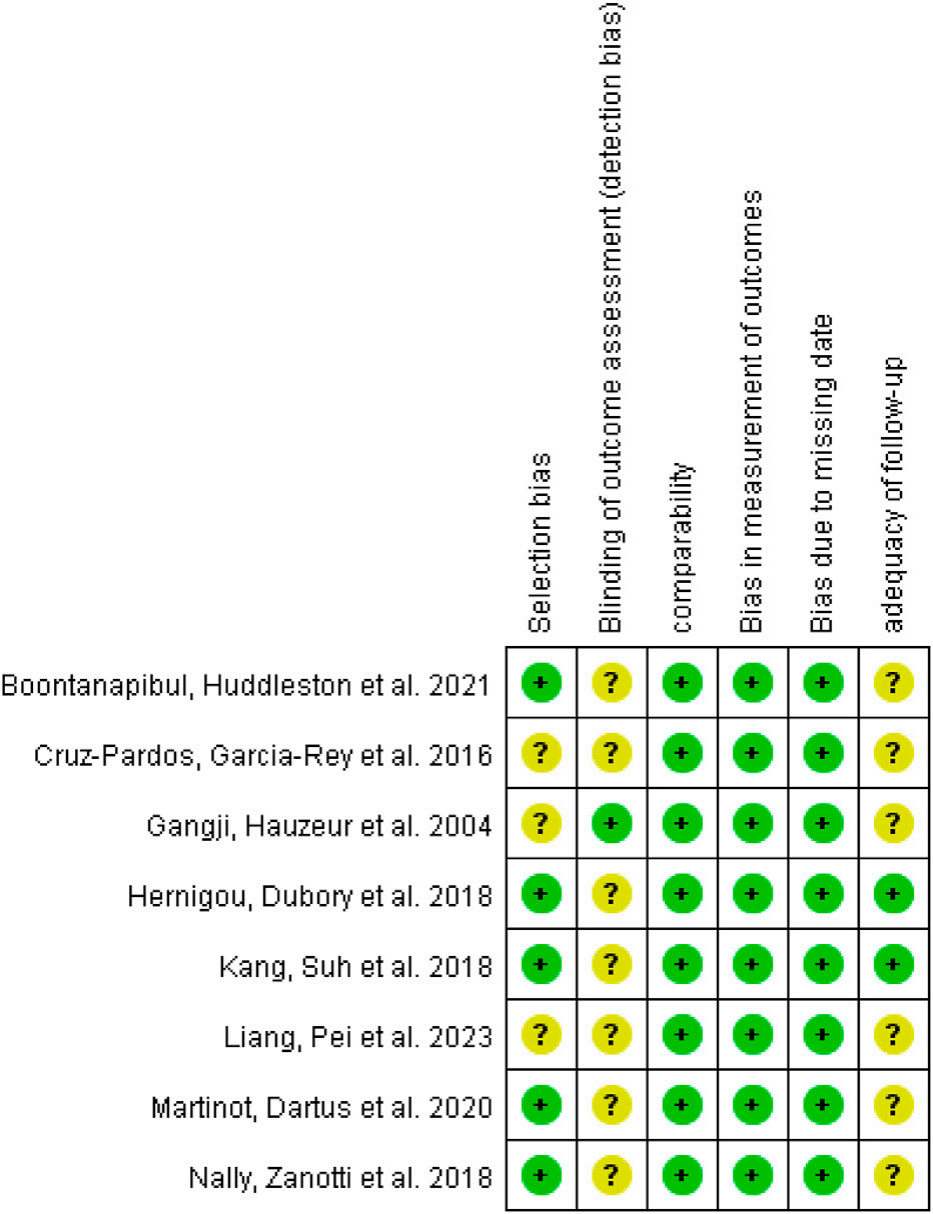
Risk of bias summary of non-RCTs: low risk of bias in green; unclear risk of bias in yellow; high risk of bias in red.

Among the enrolled RCTs, two studies explicitly described the method of randomized sequence generation (Pepke et al., 2016; Hauzeur et al., 2020), while the remaining studies did not reported on this, but based on the ROB 2.0 tool algorithm, the randomization is low risk (Gangji et al., 2011; Tabatabaee et al., 2015; Aggarwal et al., 2021; Jayankura et al., 2023; Gangji et al., 2016). Allocation concealment was implemented in three studies (Tabatabaee et al., 2015; Jayankura et al., 2023; Hauzeur et al., 2020). Blinding of participants and personnel was conducted in three studies (Gangji et al., 2011; Tabatabaee et al., 2015; Jayankura et al., 2023), with one study not employing blinding (Hauzeur et al., 2020). In terms of blinding of outcome assessment, just one study was deemed to have a unclear risk of bias and its overall risk is moderate (Tabatabaee et al., 2015). All studies provided complete outcome reports and data, with no apparent sources of bias identified. Among the non-RCTs, only one study explicitly mentioned blinding of assessors (Gangji et al., 2004), leading to an unclear risk of bias for the remaining studies (Boontanapibul et al., 2021; Cruz-Pardos et al., 2016; Kang et al., 2018; Nally et al., 2018; Liang et al., 2023; Martinot et al., 2020). Three studies had unclear selection bias (Gangji et al., 2004; Cruz-Pardos et al., 2016; Liang et al., 2023). The risk of bias summary for the included studies is presented in Figures 2, 3. One RCT (Tabatabaee et al., 2015) was deemed to be of low quality due to its uncertain overall risk and two cohort studies (Cruz-Pardos et al., 2016; Liang et al., 2023) were also considered low-quality because of more than two uncertain risk assessments.

### CD vs. CD combined with regenerative therapies outcomes

#### Stage advancement outcomes

**FIGURE 4:**
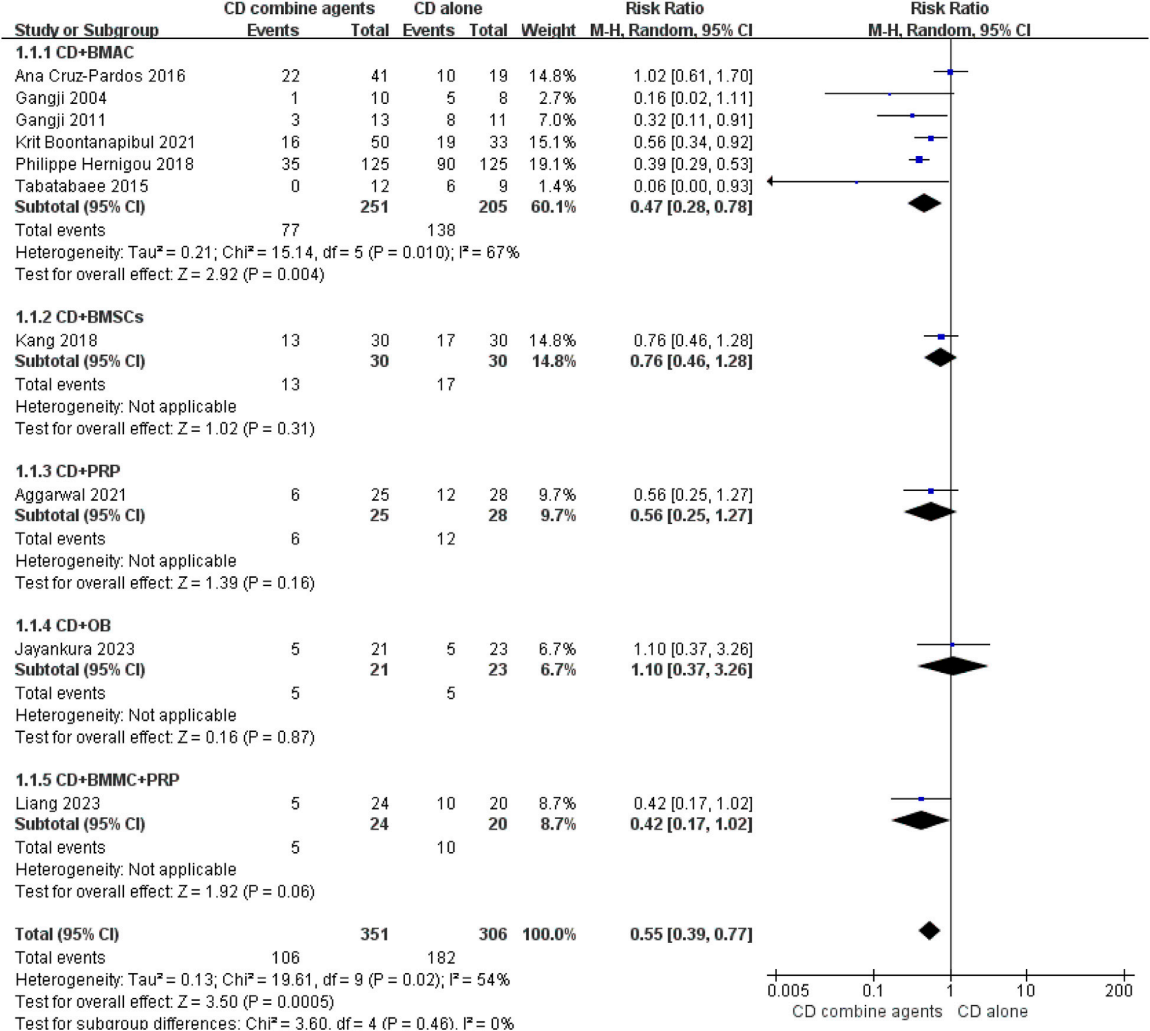
Efficacy of CD alone versus CD combined with agents. Outcome: stage progression.

**FIGURE 5:**
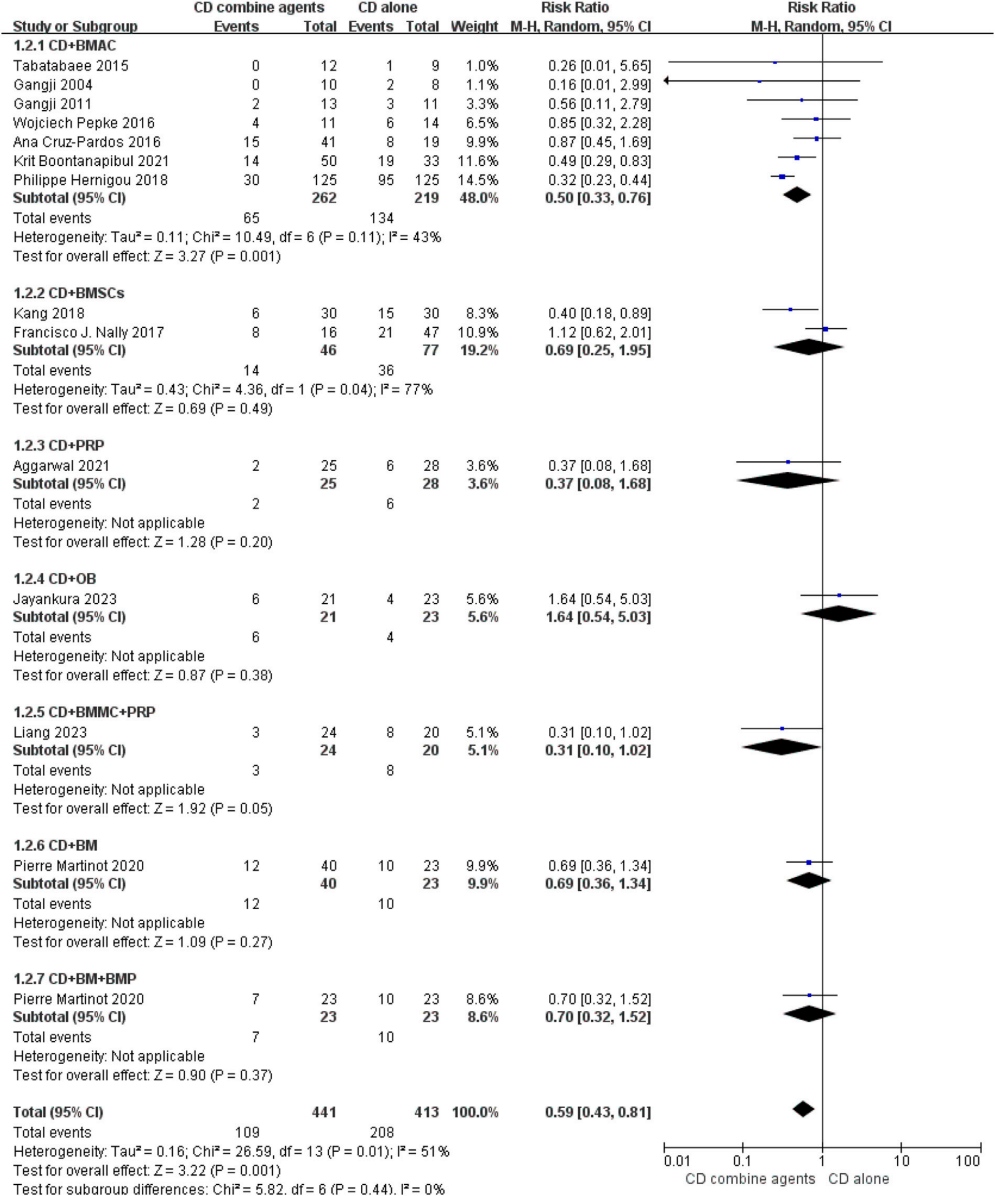
Efficacy of CD alone versus CD combined with agents, Outcome: Hip Number of THA conversion.

Ten studies reported the number of hips that progressed to collapse (Gangji et al., 2004; Boontanapibul et al., 2021; Cruz-Pardos et al., 2016; Gangji et al., 2011; Hernigou et al., 2018; Tabatabaee et al., 2015; Kang et al., 2018; Aggarwal et al., 2021; Jayankura et al., 2023; Liang et al., 2023), and 14 studies reported the number of hips that underwent THA conversion (Gangji et al., 2004; Boontanapibul et al., 2021; Cruz-Pardos et al., 2016; Gangji et al., 2011; Hernigou et al., 2018; Pepke et al., 2016; Tabatabaee et al., 2015; Kang et al., 2018; Nally et al., 2018; Aggarwal et al., 2021; Jayankura et al., 2023; Liang et al., 2023; Martinot et al., 2020). The forest plots are displayed in Figures 4, 5. The pooled analysis indicated that CD combined with regenerative therapies demonstrated a significant improvement in efficacy compared to CD alone, with both progression (risk ratio (RR) = 0.55, 95% CI 0.39 to 0.77, *p* < 0.001, *I* ^*2*^ = 54%) and THA conversion (RR = 0.59, 95% CI 0.43 to 0.81, *p* = 0.001, *I* ^*2*^ = 51%) showing statistical significance. Given the uniqueness of the two studies (Tabatabaee et al., 2015; Aggarwal et al., 2021), bone plugs were employed to seal the entrance of the pipeline to avoid the leakage of the regeneration agent, which could potentially impact the test results. Consequently, we performed a subgroup analysis regarding the use of bone suppositories, and the outcomes still indicated that CD combined with the regeneration preparation was superior to CD alone, with both progression (RR = 0.57, 95% CI 0.40 to 0.81, *p* = 0.002, *I* ^*2*^ = 59%) and THA conversion (RR = 0.60, 95% CI 0.42 to 0.87, *p* = 0.007, *I* ^*2*^ = 61%) showing statistical significance (Supplementary Figures 4, 5). Then we conducted subgroup analysis of different regenerative agents, which revealed that only the CD combined with BMAC group exhibited a statistically significant improvement in efficacy for both outcomes, with RR = 0.47 (95% CI 0.28 to 0.78, *p* = 0.004, *I* ^*2*^ = 67%) and RR = 0.41 (95% CI 0.32 to 0.52, *p* < 0.001, *I* ^*2*^ = 43%), respectively. A funnel plot indicated the presence of significant publication bias (Supplementary Figures 2, 3). After excluding the three low-quality studies, the findings continued to demonstrate that CD combined regeneration therapy was superior to CD alone, in terms of progression (RR = 0.47, 95% CI 0.38 to 0.59, *p* < 0.001, *I* ^*2*^ = 37%) and THA conversion (RR = 0.59, 95% CI 0.41 to 0.86, *p* = 0.005, *I* ^*2*^ = 57%). Subgroup analysis also showed that only the BMAC group showed a statistically significant improvement with RR = 0.40 (95% CI 0.32 to 0.52, *p* < 0.001, *I* ^*2*^ = 0%) and RR = 0.37 (95% CI 0.29 to 0.48, *p* < 0.001, *I* ^*2*^ = 23%), respectively (Supplementary Figures 6, 7).

In the subgroup analysis of CD combined with BMAC, significant differences were observed between low- and high-dose groups in the number of hips progressing to collapse with RR = 0.56 (95% CI 0.34 to 0.92, *p* = 0.020, *I* ^*2*^ = 0%) and RR = 0.23 (95% CI 0.10 to 0.56, *p* = 0.001, *I* ^*2*^ = 0%). However, only the low-dose group showed statistically significant reductions in THA conversions (RR = 0.55, 95% CI 0.35 to 0.88, *p* = 0.010, *I* ^*2*^ = 0%). After removed the low-quality studies, both the low- and medium-dose group showed satisfactory efficacy (Supplementary Figures 13, 15). Furthermore, statistically significant differences in both staging progression and THA conversion were observed when the patient population was younger than 40 years old (RR = 0.40, 95% CI 0.31 to 0.52, *p* < 0.001, *I* ^*2*^ = 44% and RR = 0.35, 95% CI 0.26 to 0.46, *p* < 0.001, *I* ^*2*^ = 0%, respectively). Although there were no statistical differences in the two subgroups in staging progression after excluding the low-quality studies, only the patients who younger than 40 years old showed significant differences in THA conversion rate (RR = 0.35, 95% CI 0.26 to 0.46, *p* < 0.001, *I* ^*2*^ = 46%) (Supplementary Figures 17, 19). In the follow-up time subgroup, a statistically significant difference in the two primary outcomes was noted when the follow-up exceeded 60 months (RR = 0.47, 95% CI 0.28 to 0.78, *p* < 0.001, *I* ^*2*^ = 0%) and 0.41 (95% CI 0.32 to 0.52, *p* < 0.001, *I* ^*2*^ = 0%). However, when considering BMI, the difference was statistically significant regardless of whether BMI exceeded 24, with an overall RR of 0.51 (95% CI 0.37 to 0.71, *p* < 0.001, *I* ^*2*^ = 0%). After sensitivity analysis, the results of these two subgroups did not change (Table 2).

#### Clinical outcomes

Seven studies reported the VAS score, revealing a statistically significant difference favoring CD combined with regenerative therapies over CD alone. The CD alone group exhibited 12.86 points higher VAS scores than the CD combined with regenerative therapies group (95% CI -18.36 to −7.36, *p* < 0.001, *I* ^*2*^ = 91%). Four studies reported the HHS score, with a statistically significant difference observed between the two groups. The CD alone group had 9.19-point lower HHS score compared to the CD combined with regenerative therapies group (95% CI 5.69 to 12.70, *p* < 0.001, *I* ^*2*^ = 72%). Four studies reported the WOMAC score, with no statistically significant difference found between CD alone and CD combined with regenerative therapies (mean difference, 8.34; 95% CI -17.66 to 0.98, *p* = 0.080, *I* ^*2*^ = 97%). However, the CD combined with BMAC group had a 10.78-point lower than the CD group, which was statistically significant (95% CI 0.47 to 21.08, *p* = 0.040, *I* ^*2*^ = 98%). The Lequesne Index was reported by two studies, both in the CD combined with BMAC group. The CD group had a 3.39-point higher Lequesne Index than the CD combined with BMAC group, with a statistically significant difference (95% CI -4.96 to −1.83, *p* < 0.001, *I* ^*2*^ = 63%). After the three low-quality studies were removed, the results remain the same as before (Table 2).

#### CD combined with BMAC vs. CD combined with OB outcomes

Two studies reported the number of staging progressions, with a statistically significant difference favoring CD combined with OB (RR = 2.29, 95% CI 1.29 to 4.06, *p* = 0.005, *I* ^*2*^ = 0%). Only one study reported the number of THA conversions, with no statistically significant difference observed (RR = 2.34, 95% CI 0.82 to 6.66, *p* = 0.110, *I* ^*2*^ = 0%). One study reported the VAS score, with no statistically significant difference found between the CD combined with BMAC group and CD combined with OB group, despite a11.00-point lower VAS score in the former (95% CI -31.75 to 9.75, *p* = 0.300, *I* ^*2*^ = 0%) (Table 3).

**TABLE 3:** Details of the outcomes of CD + BMAC vs. CD + OB.

Outcomes	No. of studies	No. of hips	Statistical method	Pooled method	*I* ^*2*^	Effect size	*p*
Staging advancement							
1 Progression to collapse	2 (Gangji et al., 2016; Hauzeur et al., 2020)	113 (56 vs. 57)	RR (95% CI)	Fix, M-H	0%	2.29 [1.29, 4.06]	0.005
2 THA conversion	1 (Hauzeur et al., 2020)	53 (26 vs. 27)	RR (95% CI)	Fix, M-H	NA	2.34 [0.82, 6.66]	0.110
Clinical outcomes							
1 VAS score	1 (Hauzeur et al., 2020)	53 (26 vs. 27)	MD (95% CI)	Fix, I-V	NA	−11.00 [−31.75, 9.75]	0.300

CD, core decompression; BMAC, bone marrow aspirate concentrate; OB, osteoblast; VAS, visual analog scale; CI, confidence interval; RR, relative risk; MD, mean difference; I–V, Inverse–Variance; M–H, Mantel–Haenszel; NA, not applicable.

## Discussion

ONFH is a prevalent condition that often leads to the dysfunction or functional loss of the hip joint in young individuals. While THA is the gold standard for treating end-stage ONFH, it is not ideal for younger individuals due to the risk of postoperative complications and limited durability (Zhao and Ma, 2020). Consequently, joint-preservation treatments are of significant value, especially for this demographic (Mont et al., 2020). However, the applicability of joint-preservation procedures is not uniform across all ONFH cases. A meta-analysis by Hua et al. highlighted the variability in success rates of CD at different stages of ONFH, with a notably low success rate of 27.44% in Ficat stage III (Hua et al., 2019). Similarly, Yuan’s mid-to long-term cohort study reported a modest success rate of 33.33% for avascular fibular grafting in ARCO stage IIIb, in contrast to the 79.49% success rate observed in stages II and IIIa (Yuan et al., 2021). These findings suggest that joint-preservation procedures may be efficacious primarily in the early or mildly collapsed stages of the disease.

The landscape of joint-preservation surgical techniques is varied and includes CD, tantalum rods implantation, and both vascularized and nonvascularized bone grafts. Despite their availability, their efficacy remains a subject of debate. Porous tantalum rods were once considered an optimal mechanical substitute post-CD due to their superior strength, fatigue resistance, and biocompatibility (Zhang et al., 2013). However, due to suboptimal success rates and an increased risk of THA-related complications upon failure, their use has been phased out (Cheng et al., 2018; Olsen et al., 2016; Shuler et al., 2007; Tanzer et al., 2008). Bone graft offer an alternative form of support, with vascularized bone grafts providing the additional benefit of promoting healing through the reestablishment of blood supply (Kim et al., 2005). A network meta-analysis conducted by Hu et al. (2023) demonstrated the effectiveness of all bone grafting treatments. Nonetheless, the high technical demands and potential harvest-site morbidities, with prevalence rates of 13%–20%, limit their widespread adoption (Houdek et al., 2017; Barla et al., 2017).

In recent years, regenerative therapies, such as BMSCs and PRP, have gained prominence (Rodeo, 2023). BMSCs contribute to regeneration through direct differentiation and paracrine effects (Chang et al., 2021), while PRP significantly enhances the concentration and release of growth and differentiation factors at the site of damage, thus accelerating the body’s natural healing process (Qian et al., 2020). These orthobiologics are straightforward to prepare and administer, prompting researchers to explore their combination with CD to enhance treatment efficacy. Li et al. (2024) conducted a network meta-analysis that subdivided regenerative agents into 6 types, revealing that only BMAC and BMSCs demonstrated superior efficacy. However, the BMAC study referenced in this article included only 245 hips and did not assess variations in BMAC dosage, hip functionality, or pain levels. Compared with this article, we further evaluated the most appropriate dose of BMAC, the age of the appropriate population, BMI, and its long-term efficacy. In addition, the sample size of the BMAC group was increased to 481 hips and the quality of life of patients was evaluated by VAS, HHS, and WOMAC scores. In two other meta-analysis, researchers also confirmed the effectiveness of cell therapy in enhancing the efficacy of CD (Li et al., 2023; Saini et al., 2023). However, in these two articles, several studies included utilized cell therapy in conjunction with bone grafts to provide mechanical support, which may compromise the comparability among studies. Furthermore, the inclusion of patients with ARCO stage III and IV in certain studies also undermined the reliability of their conclusions. In our studies, we only included patients in the early stage of ONFH, and excluded studies combined with bone grafting, which increased the reliability of the conclusion.

The collapse of the femoral head is a critical factor in determining the suitability of head preservation therapy for ONFH patients (Kuroda et al., 2019). We assessed the efficacy of specific treatment regimens using the number of hips that progressed to collapse and required THA as primary outcome indicators. A Study had suggested that CD combined with BMSCs or BMAC is more effective than CD alone (Wang et al., 2019a). Another systematic review by Han et al. published in 2020 found that PRP could improve treatment outcomes for patients with early-stage ONFH, both in combination with CD and other regimens (Han et al., 2020). However, recent research had not observed any additional benefits from combining CD with OB (Jayankura et al., 2023). Our results indicated that CD combined with BMAC group exhibited significant efficacy, with other regenerative therapies, such as BMSCs and PRP, failing to demonstrate satisfactory outcomes. The discrepancy may be attributed to the complex cellular composition of BMAC, which includes macrophages known to enhance MSC osteogenic differentiation (Kim and Hematti, 2009; Loi et al., 2016; Lu et al., 2017). The reference to BMAC as BMSCs may not be entirely accurate (Jeyaraman et al., 2021). Additionally, the limited number of studies and small sample sizes may have hindered the ability to draw accurate conclusions regarding the combination of CD with other regenerative agents. As noted previously, two articles employed bone plugs to seal the pipeline (Tabatabaee et al., 2015; Aggarwal et al., 2021), which might have an impact on the accuracy of the results. Hence, a subgroup analysis was conducted. The subgroup analysis indicated that conventional regenerative therapy still demonstrated superior efficacy. However, the efficacy of the two studies involving the addition of bone suppositories remained ambiguous, potentially due to the small sample size. Interestingly, the article using bone plugs was deleted in the sensitivity analysis of BMAC, and the results did not change significantly. Therefore, bone plugs might only serve the function of preventing the extravasation of regenerative agents, and their supporting effect could be limited. The use of bone plugs in subsequent clinical practice is controversial, as the relationship between their benefits and the risk of complications remains uncertain.

The comparison between BMAC and OB did not yield a clear advantage for OB, with a 2023 RCT recommending against their combined use in ONFH treatment due to a lack of observed benefits (Jayankura et al., 2023). Despite decreased osteoblast activity being a pathological feature of ONFH (Maestro-Paramio et al., 2021), direct supplementation of osteoblasts has not proven feasible. Unfortunately, there are few studies that directly compare the efficacy of BMAC with other regenerative agents. Although they possess certain capacity for tissue repair, the majority of regenerating agents are not directly replenished by BMSCs, which may contribute to their failure. Thus, BMAC is considered a reliable regenerative therapy, but further trials are needed to confirm the effectiveness of other regenerative agents. Limitations in BMAC include variability in preparation techniques and a lack of consensus on optimal dosages. The ideal patient population for BMAC treatment remains undefined, as host factors such as age and BMI can influence the efficacy of regenerative therapeutics (Mao et al., 2020; Pan et al., 2020). To better define the optimal population and treatment efficacy, we conducted subgroup analyses based on BMAC dosage, age, BMI, and follow-up duration.

Given that the average necrotic volume for stage III femoral head necrosis is approximately 22 mL Hu et al. (2015), the injected dose should ideally be less than this volume to avoid increased pressure in the bone marrow cavity and potential leakage Our analysis stratified the BMAC dosage into three groups based on 20 mL increments. A study published latest suggested that a 20 mL BMAC dosage may yield better results (Wang et al., 2023), but it its inclusion of bone grafts alongside other studies introduces a confounding factor, leading to less robust conclusions. Our findings indicate that the low-dose BMAC group (less than 20 mL) exhibited a more favorable effect in mitigating the progression of femoral head necrosis. The determination of the optimal BMAC dosage necessitates further trials, and we propose that administering less than 20 mL of BMAC may be a prudent strategy. Moreover, MSC function is influenced by numerous factors, including the obese environment and host age, with a reduction in MSC function and quantity observed in both obese and older patients (Carvalho et al., 2021; Pham et al., 2023). A meta-analysis has identified patients under 40 years of age as an ideal population for stem cell treatments (Mao et al., 2020). Studies have also implicated a BMI exceeding 30 as an independent risk factor for imaging progression and THA conversion following CD plus BMAC (Hoogervorst et al., 2022). with a BMI over 24 conferring a 2.58-fold increased risk of joint preservation failure compared to patients with a BMI under 24 (Pan et al., 2020). Our subgroup analysis of age and BMI corroborated the enhanced efficacy of CD plus BMAC in patients under 40. However, no significant efficacy difference was noted for patients with a BMI over 24, possibly due to the majority of patients having a BMI above 24 but not meeting the obesity threshold (BMI >28), and the small sample size’s potential influence on the outcomes. A study by Hernigou et al. (2018) with a follow-up of up to 25 years, demonstrated that CD plus BMAC improved disease prognosis (Osawa et al., 2021). Our findings align with this conclusion, although the efficacy of CD plus BMAC did not differ significantly in studies with follow-up periods of less than 24 months. This may indirectly suggest that CD alone has improved the near-term prognosis of femoral head necrosis, offering a modestly meaningful effect.

PRP is a concentrated preparation of autologous plasma that not only encompasses a diverse array of growth factors conducive to bone induction and tissue regeneration, but also effectively eliminates inflammatory mediators to alleviate pain. Its elevated concentration of growth factors can stimulate the proliferation of osteoblasts and chondrocytes, while also facilitating neovascularization in necrosis regions to enhance blood supply. Consequently, PRP may represent a promising therapeutic approach for the management of ONFH (Su et al., 2022; Tong et al., 2018). However, the literature on PRP’s use in ONFH is scarce, with only a few studies suggesting its potential in delaying ONFH progression. The small sample sizes in these studies preclude the drawing of definitive conclusions. Moreover, similar to BMAC, the lack of detailed reporting on PRP formulations hampers a comprehensive assessment of its efficacy (Chahla et al., 2017). Further research is imperative to establish the role of PRP in ONFH, with a need for clarification on PRP compositions. Osteoblasts play a crucial role in the formation of new bone, as they not only directly promote osteogenesis but also modulate osteoclast activity and angiogenesis in areas of necrosis (Chen et al., 2018). Preliminary studies have indicated that enhancing osteoblast function via upstream signaling pathways can ameliorate femoral head necrosis (Wang et al., 2019b; Tian et al., 2020). However, clinical applications remain relatively constrained. Future research ought to focus on conducting high-quality, large-scale studies to validate the efficacy of regenerative agents.

In terms of hip pain and function, our study’s findings, in concordance with the majority of previous studies (Wang et al., 2023; Wang et al., 2019a), indicate that the combination of CD with regenerative therapies significantly improves hip pain and function metrics (VAS, HHS, WOMAC, and Lequesne Index). This suggests that the integration of regenerative therapies with CD is likely effective in enhancing hip pain and function. However, the lack of statistical significance in certain subgroups may be attributable to small sample sizes. A 2020 study by Hauzeur et al. comparing VAS scores between CD combined with BMAC and CD combined with OB found no significant difference, implying that OB does not offer additional benefits over BMAC in terms of hip pain and function (Hauzeur et al., 2020).

Besides, we performed a sensitivity analysis due to the heterogeneity of this study. There were no significant changes in the results of the two primary outcome measures and various hip functional scores after excluding the three low-quality studies. In the BMAC subgroup, after sensitivity analysis, the heterogeneity of the study was reduced but the conclusions did not change, indicating the reliability of the results. The only difference is that, following sensitivity analysis, moderate doses of BMAC also appear to exhibit some therapeutic effectiveness. This may be attributed to the limited number of studies, leading to conflicting results. Therefore, the optimal therapeutic dose of BMAC remains uncertain, but small doses of BMAC (less than 20 mL) are seemingly recommended.

In terms of safety, postoperative complications were reported in eight of all the included studies (Gangji et al., 2004; Gangji et al., 2011; Pepke et al., 2016; Tabatabaee et al., 2015; Jayankura et al., 2023; Martinot et al., 2020; Hauzeur et al., 2020; Fu et al., 2022). Among 311 hip joints, 26 patients expressed complaints of pain (8.36%), 2 patients presented with hematoma (0.64%), 8 patients experienced transient fever (2.57%), 2 patients had positive bone marrow bacterial culture but no infection (0.64%), and 1 patient suffered from postoperative fracture (0.32%). And there was no significant difference between the CD group and the CD combined regeneration therapy group. Additionally, among a total of 88 cases of hip joint in the BMAC-related study, 3 patients experienced pain (3.41%), 2 patients had positive bone marrow bacteriological culture but were not infected (2.27%), and 2 patients had hematoma (2.27%). These results indicate that both CD combined regenerative therapies and CD + BMAC possess a considerable safety profile, at least without elevating the risk of postoperative complications related to CD alone.

The strength of our meta-analysis lies in its comprehensive comparison of different regenerative therapies based on all available RCTs and cohort studies, providing an evidence-based foundation for the application of CD combined with regenerative therapies. Nevertheless, several limitations exist. The scarcity of research on CD combined with regenerative therapies other than BMAC prevents definitive conclusions. Variations in the type of regenerative agents, regenerative therapy preparation method, dose, and cell count, as well as large differences in the number of patients and baseline data from each study, make this study highly heterogeneous and may affect the reliability of the results, even though using random effects model. Future trials should standardize these variables. Additionally, the lack of data precluded an assessment of prognostic factors such as preoperative femoral head necrosis volume, etiologies, and gender. Subgroup analyses of these factors are necessary to guide clinical applications effectively. More clinical trials are warranted to identify independent risk factors affecting the prognosis of CD in early-stage ONFH patients and to further refine clinical practices. Finally, because there are few studies with long-term follow-up, relatively short follow-up times can also make conclusions unreliable. Longer follow-up studies are needed.

## Conclusion

Our analysis of various randomized controlled trials and cohort studies suggests the combination of CD and regenerative therapies, particularly BMAC, can enhance pain relief and functional improvement in ONFH patients. However, current evidence suggests that only BMAC shows potential to delay the progression of ONFH. A low dosage of BMAC (less than 20 mL) for patients who under 40 may yield the most considerable efficacy. Similar future studies should focus on longer follow-up durations, the comparative analysis of efficacy variations across different doses or cell concentrations of regenerative agents, and the establishment of standardized procedures for regenerative preparation.

## Abbreviations

ONFH, osteonecrosis of the femoral head; THA, total hip arthroplasty; CD, core decompression; BMAC, bone marrow aspirate concentrate; BMPs, bone morphogenetic proteins; PRP, platelet-rich plasma; BMSCs, bone mesenchymal stem cells; OB, osteoblasts; VAS, visual analog scale; HHS, Harris Hip Score; WOMAC, Western Ontario and McMaster Universities Osteoarthritis Index.
