# Supplementary material for: The efficacy of core decompression combined with regenerative therapy in early femoral head necrosis: a systematic review and meta-analysis involving 954 subjects
Source: Front Pharmacol. 2025 Jan 7;15:1501590. doi: 10.3389/fphar.2024.1501590 (PMC11747542; doi:10.3389/fphar.2024.1501590)
Supplement: Supplementary file 1 [file DataSheet1.docx]

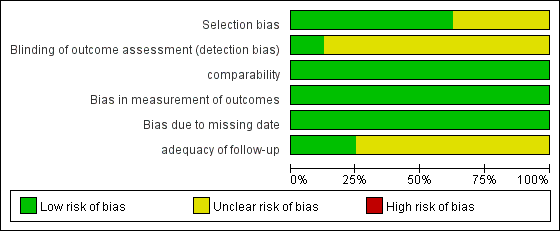


**SFigure 1** Risk of bias graph: low risk of bias in green; unclear risk of bias in yellow; high risk of bias in red.


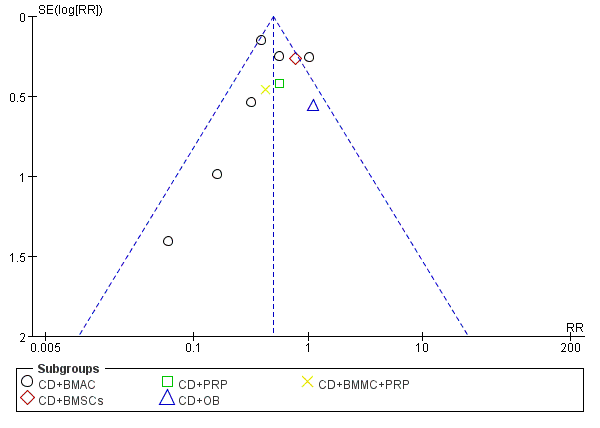


**SFigure 2** Funnel plot of publication bias for the included trials comparing the number of progression collapse between CD and CD combined with agents.


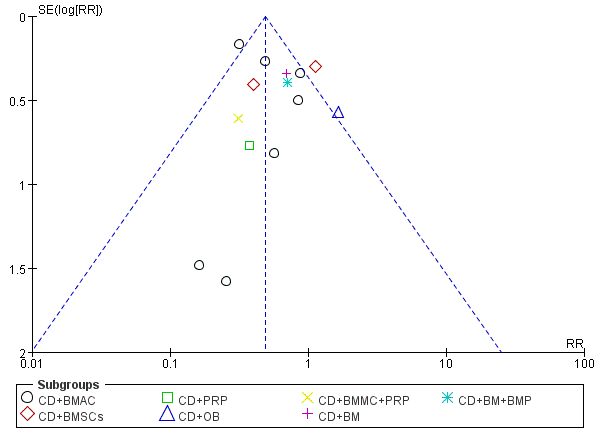


**SFigure 3** Funnel plot of publication bias for the included trials comparing the number of THA conversions between CD and CD combined with agents.


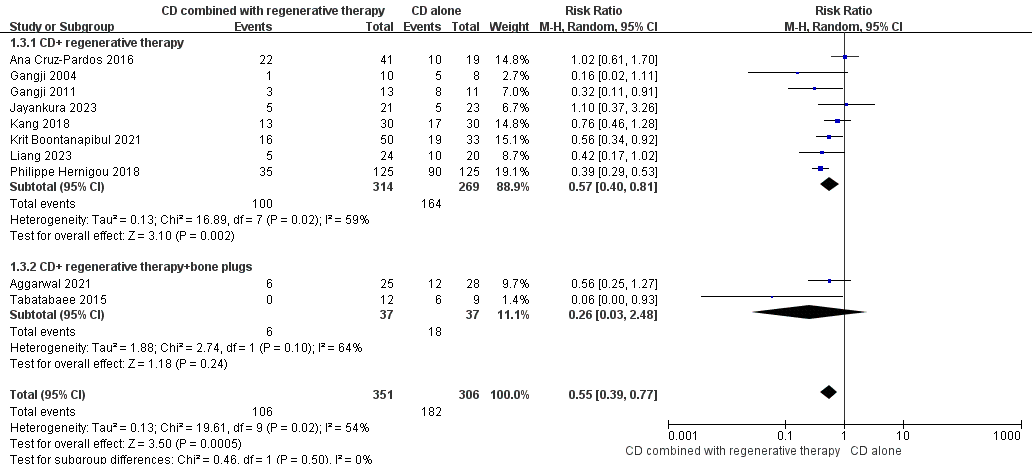


**SFigure 4** Efficacy of CD alone versus CD combined with agents. Outcome: stage progression.

**
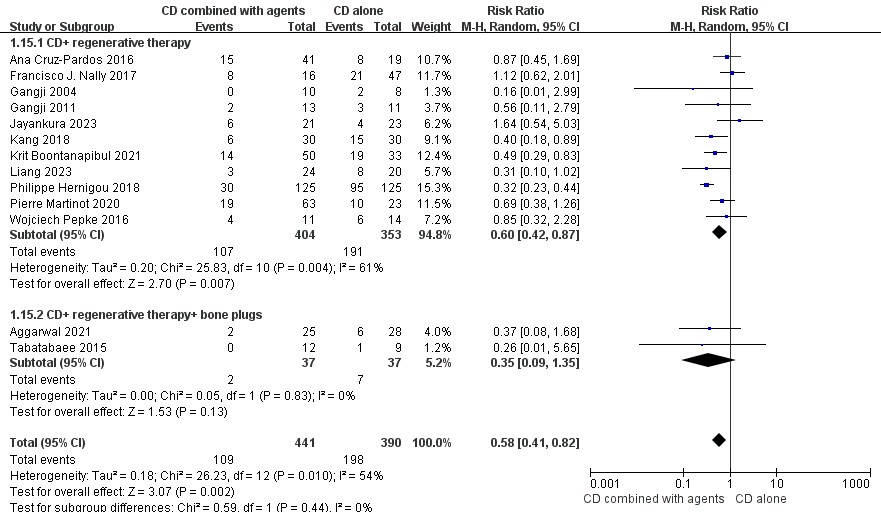
**

**SFigure 5** Efficacy of CD alone versus CD combined with agents. Outcome: Number converted to THA.


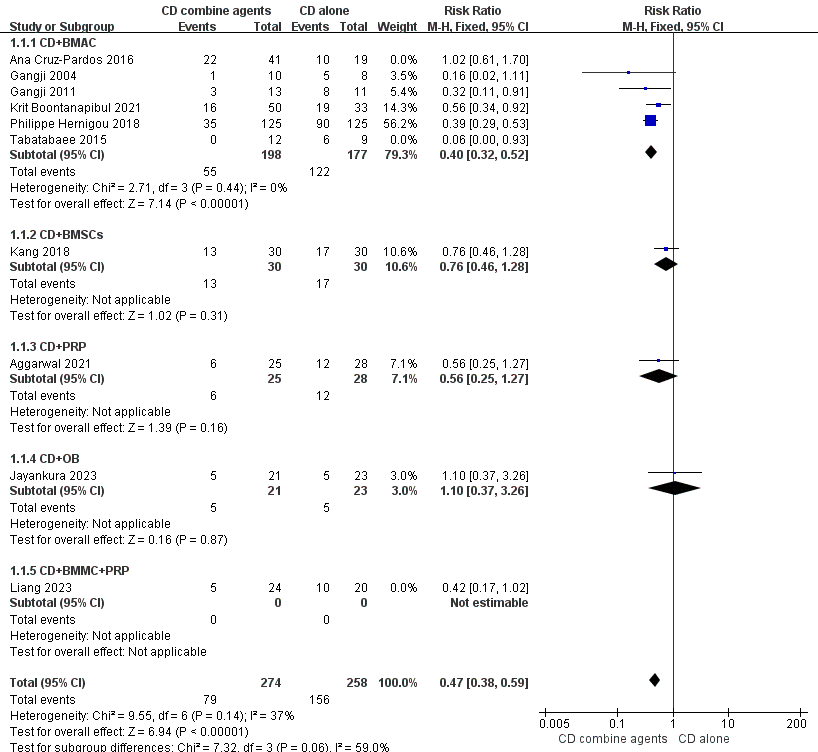


**SFigure 6** Efficacy of CD alone versus CD combined with agents. Outcome: stage progression (after removed three low quality studies).


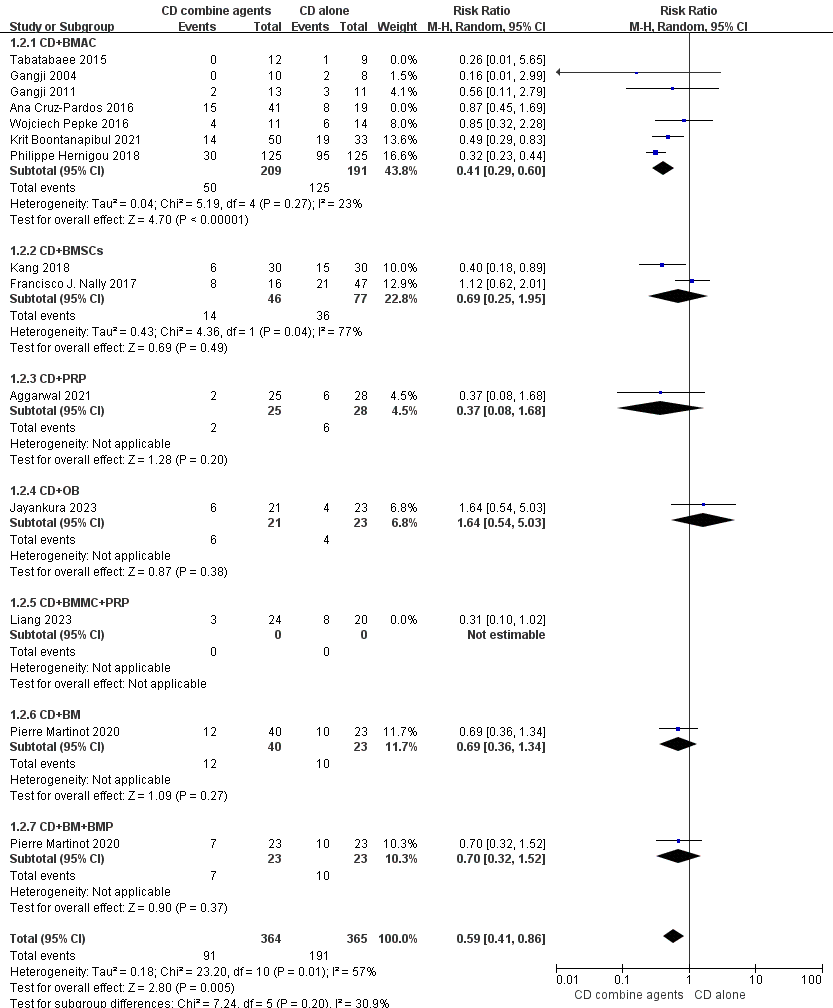


**SFigure 7** Efficacy of CD alone versus CD combined with agents. Outcome: Hip Number of THA conversion (after removed three low quality studies).


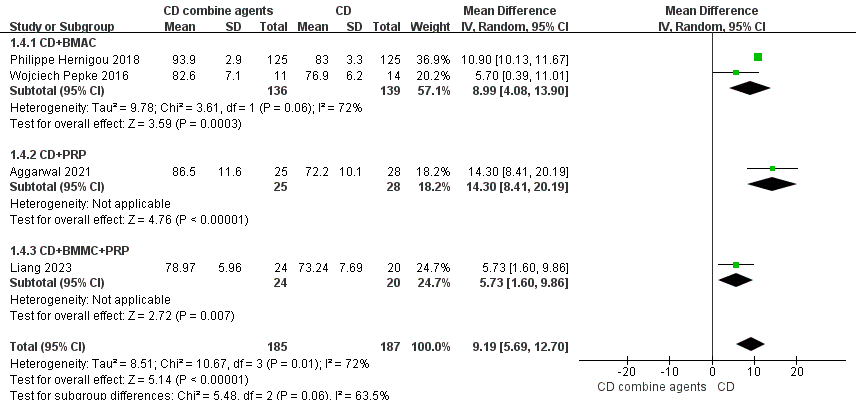


**SFigure 8** Efficacy of CD alone versus CD combined with agents. Outcome: HHS scores

**
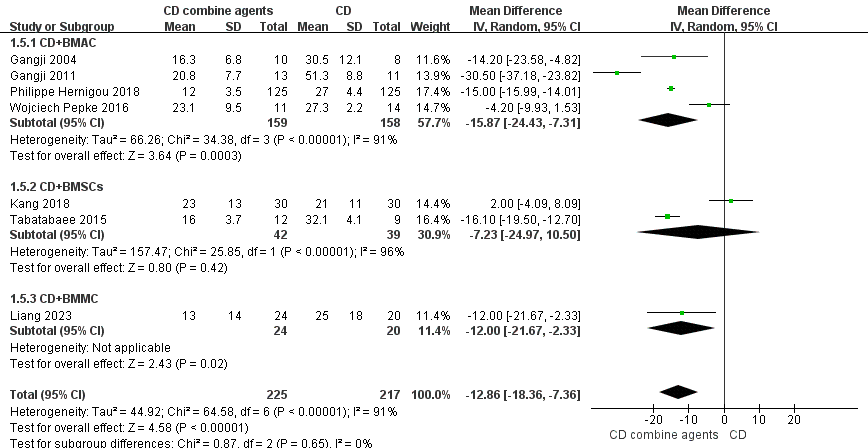
**

**SFigure 9** Efficacy of CD alone versus CD combined with agents. Outcome: VAS scores

**
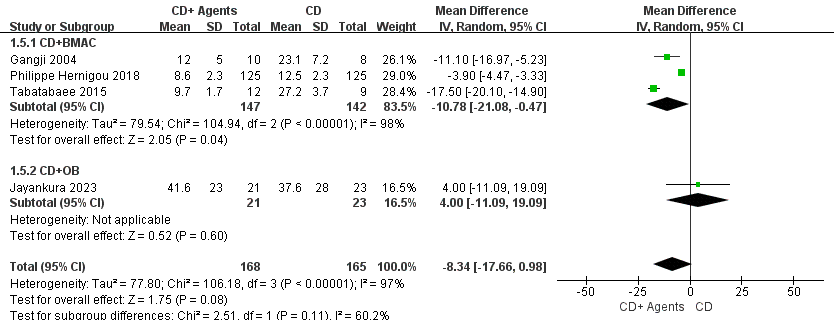
**

**SFigure 10** Efficacy of CD alone versus CD combined with agents. Outcome: WOMAC scores

**
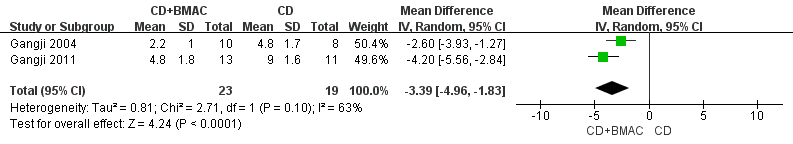
**

**SFigure 11** Efficacy of CD alone versus CD combined with agents. Outcome: Lequesne Index

**
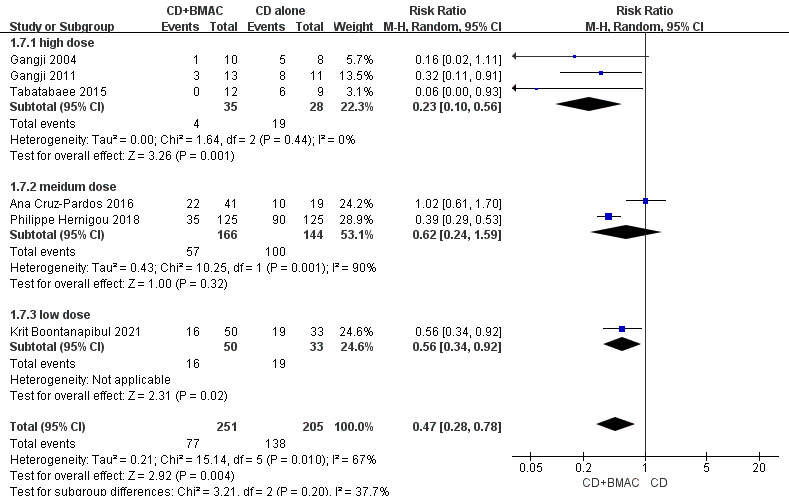
**

**SFigure 12** Efficacy of CD alone versus CD combined with different BMAC dosesage. Outcome: stage progression


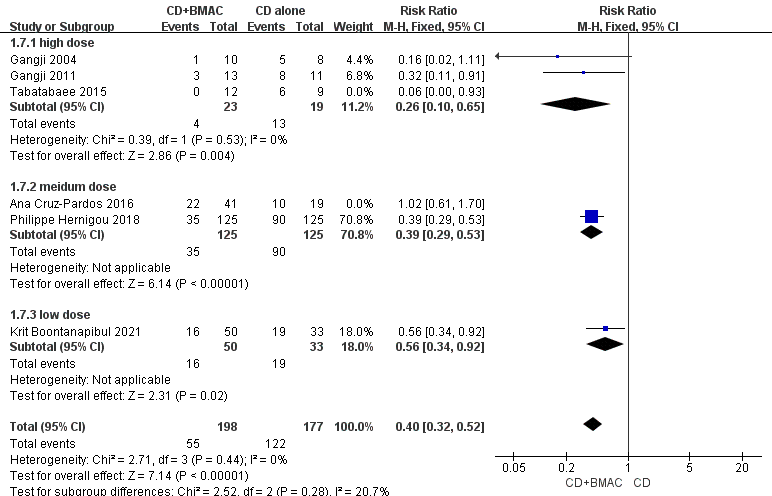


**SFigure 13** Efficacy of CD alone versus CD combined with different BMAC dosage. Outcome: stage progression (after removed three low quality studies).


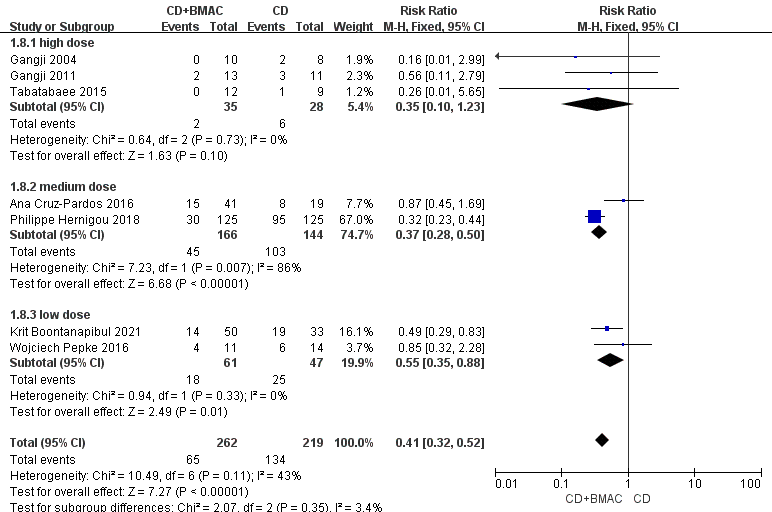


**SFigure 14** Efficacy of CD alone versus CD combined with different BMAC dosage. Outcome: THA conversion


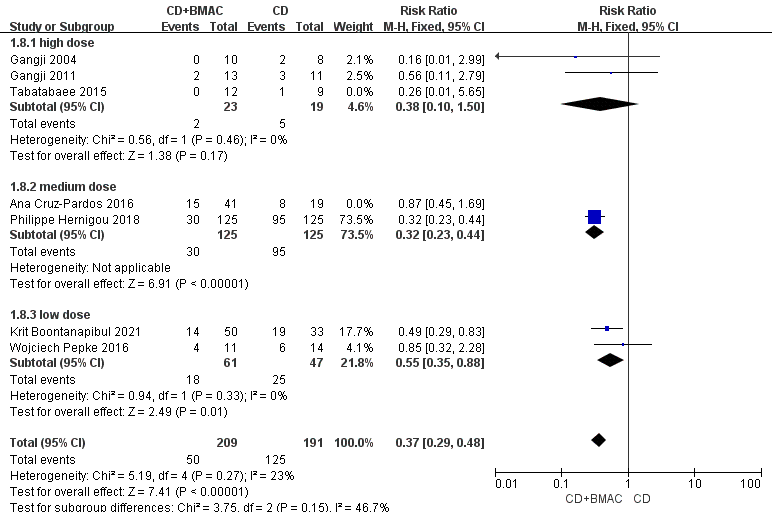


**SFigure 15** Efficacy of CD alone versus CD combined with different BMAC dosage. Outcome: THA conversion (after removed three low quality studies).


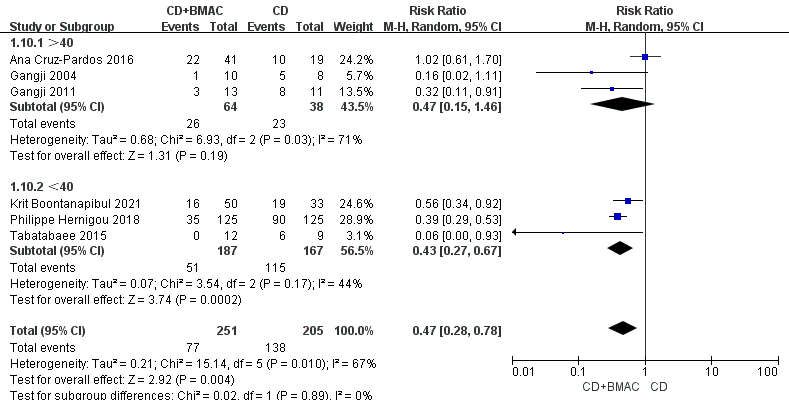


**SFigure 16** Efficacy of CD alone versus CD combined with different BMAC in different age groups. Outcome: stage progression


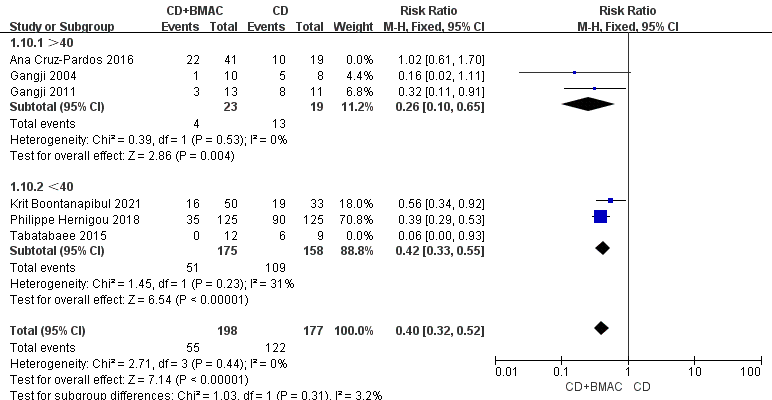


**SFigure 17** Efficacy of CD alone versus CD combined with different BMAC in different age groups. Outcome: stage progression (after removed three low quality studies).

**
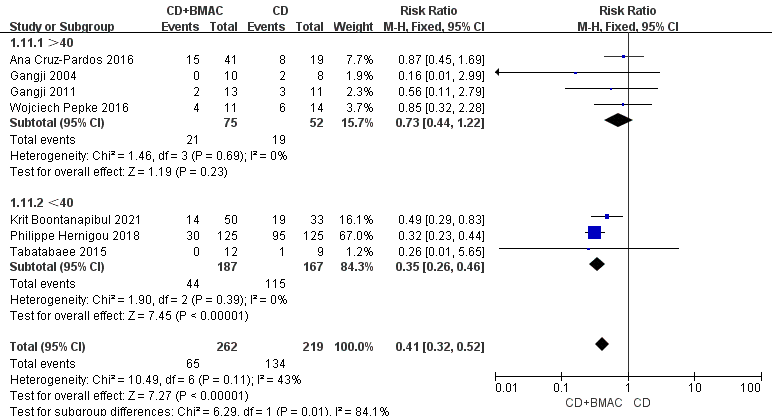
**

**SFigure 18** Efficacy of CD alone versus CD combined with different BMAC in different age groups. Outcome: THA conversion.


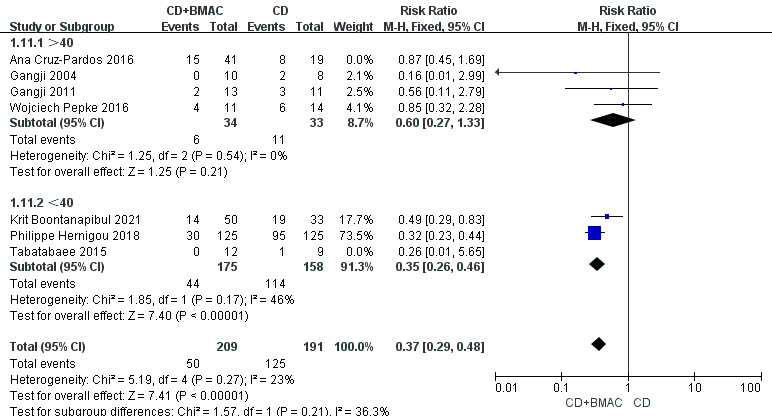


**SFigure 19** Efficacy of CD alone versus CD combined with different BMAC in different age groups. Outcome: THA conversion (after removed three low quality studies).

**
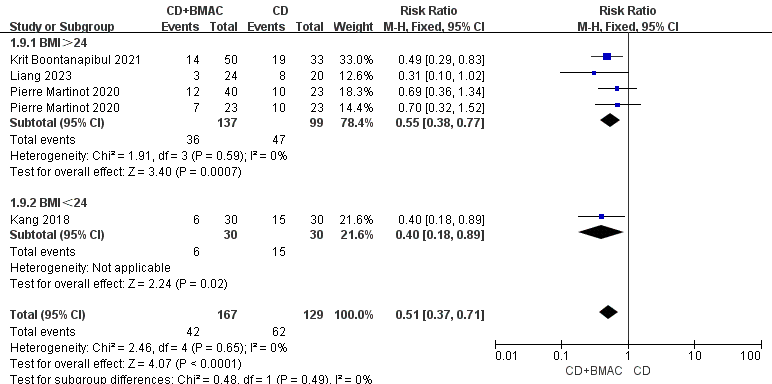
**

**SFigure 20** Efficacy of CD alone versus CD combined with different BMAC in different BMI groups. Outcome: THA conversion


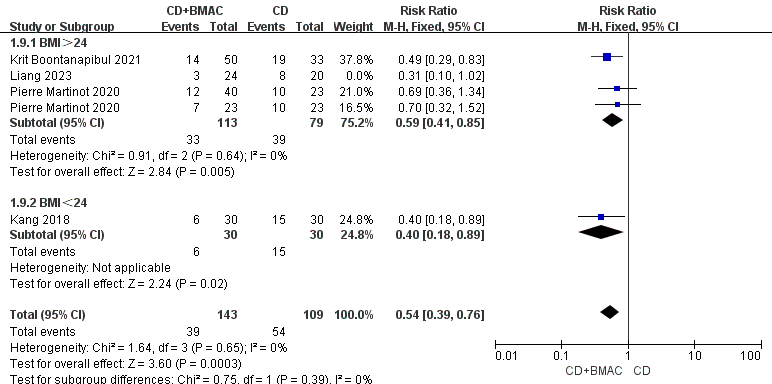


**SFigure 21** Efficacy of CD alone versus CD combined with different BMAC in different BMI groups. Outcome: THA conversion (after removed three low quality studies).

**
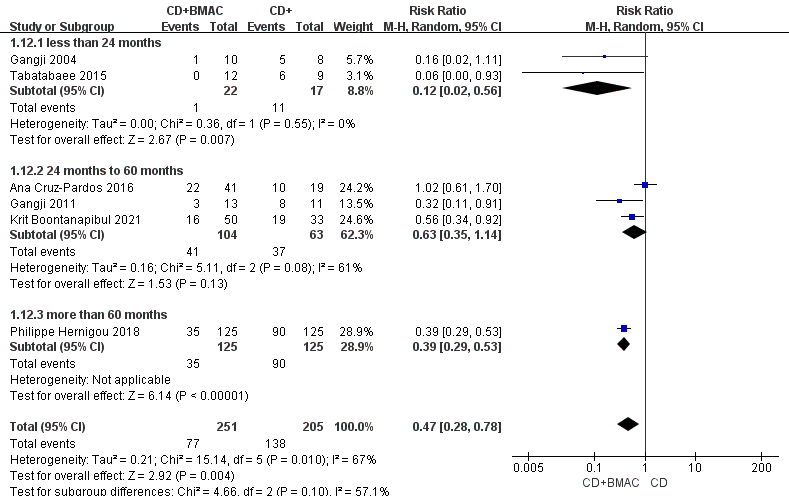
**

**SFigure 22** Efficacy of CD alone versus CD combined with BMAC in different follow-up time groups. Outcome: stage progression


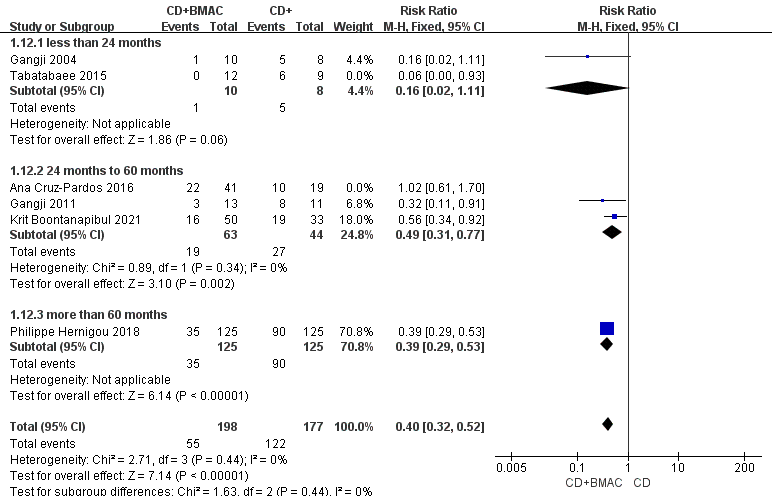


**SFigure 23** Efficacy of CD alone versus CD combined with BMAC in different follow-up time groups. Outcome: stage progression (after removed three low quality studies).

**
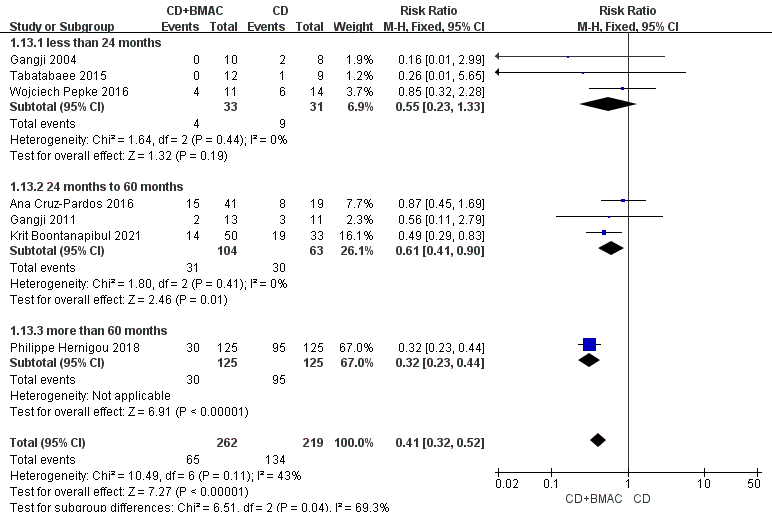
**

**SFigure 24** Efficacy of CD alone versus CD combined with BMAC in different follow-up time groups. Outcome: THA conversion


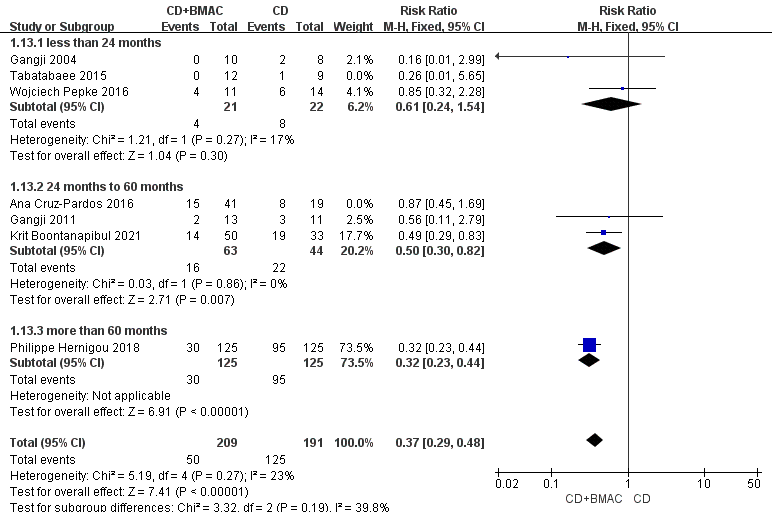


**SFigure 25** Efficacy of CD alone versus CD combined with BMAC in different follow-up time groups. Outcome: THA conversion (after removed three low quality studies).

**
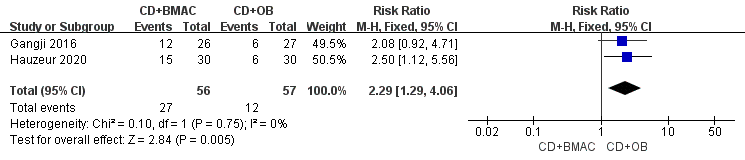
**

**SFigure 26** Efficacy of CD+ BMAC versus CD+ OB. Outcome: stage progression

**
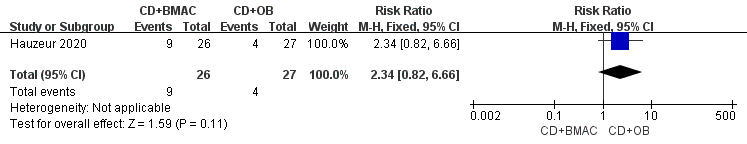
**

**SFigure 27** Efficacy of CD+ BMAC versus CD+ OB. Outcome: THA conversion

**
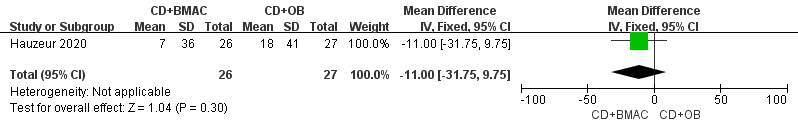
**

**SFigure 28** Efficacy of CD+ BMAC versus CD+ OB. Outcome: VAS scores
